# Shared genetics between ADHD and reading/language abilities: Genome‐wide correlations, stratified enrichment, cross‐trait association, and mendelian randomization

**DOI:** 10.1002/jcv2.70148

**Published:** 2026-07-18

**Authors:** Jinzhu Zhao, Shi Huang, Wei Liu, Feng Wang, Hong Qian, Xiaolin Hu

**Affiliations:** ^1^ Division of Child Healthcare Department of Pediatrics Tongji Hospital Tongji Medical College Huazhong University of Science and Technology Wuhan China; ^2^ Department of Public Health Tongji Hospital Tongji Medical College Huazhong University of Science and Technology Wuhan Hubei China; ^3^ Tongji Medical College Huazhong University of Science and Technology Wuhan Hubei China

**Keywords:** ADHD, genetic correlation, GWAS, Mendelian randomization, reading‐ and language‐related skills

## Abstract

**Background:**

Attention‐deficit/hyperactivity disorder (ADHD) and language/reading difficulties frequently co‐occur. The extent of shared genetic architecture remains incompletely defined. We investigated genome‐wide overlap between ADHD and four core skills: word reading, nonword reading, spelling, and phoneme awareness.

**Methods:**

We integrated genome‐wide association study (GWAS) summary statistics from the Psychiatric Genomics Consortium for ADHD (38,691 cases; 186,843 controls) and from the GenLang Consortium for language‐related traits (*n* = 13,633–33,959). We performed linkage disequilibrium score regression (LDSC) to estimate genetic correlations and stratified LDSC to identify enriched genomic annotations. Cross‐trait meta‐analyses were conducted using MTAG and CPASSOC to identify cross‐trait association signals. Mendelian randomization (MR) was applied to assess potential directional relationships.

**Results:**

Genome‐wide genetic correlations were negative and significant between ADHD and each reading‐ and language‐related trait (word reading rg = −0.35, *p* = 1.35 × 10^−16^; nonword reading rg = −0.28, *p* = 2.15 × 10^−9^; spelling rg = −0.38, *p* = 1.03 × 10^−15^; phoneme awareness rg = −0.28, *p* = 2.64 × 10^−6^). Partitioned heritability analysis using S‐LDSC showed significant enrichment after Benjamini‐Hochberg false discovery rate correction (BH‐FDR), concentrated in conserved or constrained annotations such as Genomic Evolutionary Rate Profiling and phastCons. Cross‐trait analyses (MTAG and CPASSOC) identified loci associated with ADHD and each skill, 6 for word reading, 7 for spelling, 7 for phoneme awareness, and 4 suggestive loci for nonword reading (dual‐method *p* < 5 × 10^−6^); several map near neurodevelopmental genes including DCC, MEF2C, and ST3GAL3. Bidirectional MR findings were consistent with a potential directional contribution of ADHD genetic liability to poorer reading/language performance, with no clear evidence supporting the reverse direction under standard MR assumptions.

**Conclusion:**

ADHD and language/reading abilities share a substantial polygenic overlap with convergent signals in neuronal regulatory annotations. These genome‐wide findings support a transdiagnostic framework linking attention and literacy‐related skills, while indicating that causal and clinical interpretations should remain cautious given the modest effect sizes and limited variance captured by current GWAS resources.

## Key Points

**What's known?**

- ADHD frequently co‐occurs with reading‐/language‐related difficulties, and twin studies suggest partly shared genetic influences.

**What's new?**

- This genome‐wide cross‐trait analysis found significant negative genetic correlations between ADHD and word reading, nonword reading, spelling, and phoneme awareness.
- Cross‐trait analyses identified shared signals near neurodevelopmental genes, including DCC, MEF2C, and ST3GAL3.
- Mendelian randomization results were consistent with a modest potential directional association between ADHD genetic liability and poorer reading‐/language‐related performance.

**What's relevant?**

- These findings support integrated assessment of attentional and literacy‐related difficulties, while causal and clinical interpretations should remain cautious.

## INTRODUCTION

Human language and reading ability are complex neurocognitive functions central to communication and learning (Fisher & Marcus, 2006). Twin and family studies have shown that reading‐ and language‐related skills are moderately to highly heritable, with twin‐based heritability estimates commonly ranging from approximately 30%–80%, depending on the specific phenotype, age, language, and measurement approach (Andreola et al., 2021; Eising et al., 2022; Little et al., 2017). These genetic influences extend to phonological and orthographic processing, which are central components of literacy development (Church et al., 2023; Fisher et al., 1999). Recent genome‐wide association studies (genome‐wide association study (GWAS)) have begun to identify loci associated with these traits, implicating genes involved in neuronal development and synaptic signaling, such as ROBO2 and MIR924HG, which influence early vocabulary and rapid naming (Gialluisi et al., 2019; St Pourcain et al., 2014; Truong et al., 2019). However, GWAS effect sizes are typically small, and SNP‐based heritability captures only part of the heritability estimated from twin studies, indicating that much of the genetic contribution to reading‐ and language‐related skills remains unresolved (Gialluisi et al., 2021; Kovas et al., 2007; Olson et al., 2014). This unresolved heritable signal may include additional polygenic influences shared with other neurodevelopmental domains.

Attention‐deficit/hyperactivity disorder (ADHD) is one of the most prevalent neurodevelopmental disorders, affecting approximately 5%–7% of children worldwide and often persisting into adulthood (Posner et al., 2020; Thomas et al., 2015). ADHD is also highly heritable, with twin studies estimating that genetic factors account for approximately 70%–80% of phenotypic variation, and a recent review reporting a mean heritability estimate of about 74% across twin studies (Faraone & Larsson, 2019). At the molecular level, ADHD has a highly polygenic architecture involving many common variants of small effect, with GWAS and pathway analyses implicating dopaminergic, synaptic, and broader neurodevelopmental signaling (Hongyao et al., 2023; Demontis et al., 2023). Convergence with language and reading is suggested by behavioral and cognitive evidence: difficulties in phonological processing, working memory, and executive control, domains relevant to attention regulation and reading acquisition, are frequently observed in ADHD (De Groot et al., 2017; Denton et al., 2020; Li et al., 2023; Willcutt et al., 2005). Co‐occurrence is also common; approximately 20%–40% of children with ADHD have reading disability (RD), and a comparable proportion of children with RD exhibit ADHD symptoms (Willcutt et al., 2005). Importantly, twin and family studies suggest that this co‐occurrence is partly attributable to shared genetic influences rather than to phenotypic association alone. Recent twin and sibling analyses, including the study by van Bergen et al., showed that ADHD symptoms, dyslexia, spelling difficulties, and related learning traits are correlated in childhood and that their co‐occurrence can be explained substantially by overlapping genetic liabilities, with limited support for a simple trait‐to‐trait causal pathway from ADHD to reading difficulties or vice versa (van Bergen et al., 2025). These findings indicate that ADHD‐reading/language comorbidity may arise from shared inherited neurodevelopmental vulnerabilities, particularly involving attentional control, executive function, phonological processing, and literacy acquisition. Deficits in phonological working memory and central executive functioning may further contribute to reduced attention span, task persistence, and reading fluency (Alt et al., 2022; Tosto et al., 2021), leading to compounded learning difficulties and poorer academic outcomes when both conditions co‐exist (Germanò et al., 2010). At the gene and network level, loci and pathways involved in language circuit formation and cortical connectivity, including FOXP2‐related mechanisms, axon guidance genes such as DCC, and activity‐dependent regulators such as MEF2C, have been implicated in language development and reported in ADHD‐related studies, suggesting possible biological overlap without establishing locus‐level sharing (Ciulkinyte et al., 2025; Meyer et al., 2023).

A relevant framework for interpreting this overlap is the ‘generalist genes’ hypothesis, which proposes that many genetic influences on neurodevelopmental and learning difficulties are domain‐general rather than disorder‐ or skill‐specific. From this perspective, ADHD and reading‐/language‐related difficulties may partly share genetic liability through broad neurodevelopmental processes, including attention regulation, executive control, processing speed, and working memory. At the same time, domain‐specific genetic influences may contribute to phonological, orthographic, and linguistic variation. This distinction between domain‐general and domain‐specific effects provides a conceptual basis for examining whether ADHD and reading‐/language‐related traits share genome‐wide genetic correlations, functional enrichments, and cross‐trait association signals (Plomin & Kovas, 2005).

As shown in the original GenLang study by Eising et al. (2022), the four reading‐ and language‐related traits share substantial genetic architecture. However, they also represent partially distinct components of literacy and language‐related ability. Word reading primarily reflects familiar word recognition and orthographic knowledge; nonword reading indexes phonological decoding; spelling involves orthographic encoding and phoneme‐grapheme mapping; and phoneme awareness indexes metalinguistic sensitivity to speech‐sound structure. Therefore, analyzing these traits separately, rather than only relying on the shared genetic architecture across them, allows us to examine whether the genetic overlap with ADHD is broadly shared across literacy‐related skills or varies by component process. This approach provides added value by identifying both domain‐general overlap across reading‐/language‐related abilities and potential trait‐specific patterns that may be obscured in a single common‐factor analysis.

Building on this background, we investigate the shared genetic architecture between ADHD and four core reading‐related skills, word reading, nonword reading, spelling, and phoneme awareness, using large‐scale GWAS resources, including the GenLang Consortium and publicly available datasets. Specifically, we (1) quantify genome‐wide genetic correlations, (2) test for pleiotropic signals contributing to both phenotypes, and (3) evaluate potential directional relationships between ADHD liability and reading‐/language‐related outcomes using Mendelian randomization. Clarifying this overlap may refine etiological models of ADHD and its comorbid reading‐/language‐related difficulties.

## METHODS

**FIGURE 1:**
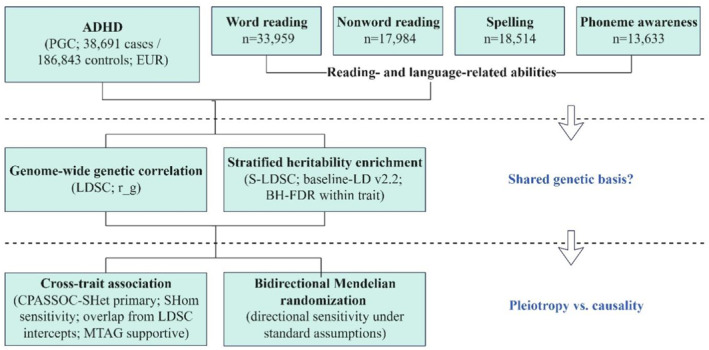
Analysis workflow for shared genetics between ADHD and reading‐/language‐related abilities (LDSC, S‐LDSC, CPASSOC/MTAG, and MR) MR, Mendelian randomization.

Figure 1 summarizes the analytic workflow. We used GWAS summary statistics for ADHD from the Psychiatric Genomics Consortium (PGC) (Demontis et al., 2023; 38,691 cases and 186,843 controls, European ancestry) (Demontis et al., 2023). Reading‐ and language‐related traits were obtained from the GenLang Consortium meta‐analyses across 22 cohorts (Eising et al., 2022): word reading (*n* = 33,959), nonword reading (*n* = 17,984), spelling (*n* = 18,514), and phoneme awareness (*n* = 13,633). Public releases were harmonized to GRCh37; we performed cross‐study alignment of effect alleles, removed multi‐allelic and palindromic SNPs, excluded the MHC region (chr6:25–34 Mb), and retained variants present in all relevant datasets. The ADHD GWAS was based on European‐ancestry participants. The GenLang summary statistics used in this study corresponded to the full GenLang meta‐analysis of quantitatively assessed reading‐ and language‐related traits, with sample sizes ranging from 13,633 to 33,959 participants. According to the original GenLang study by Eising et al. (2022), most participants were of European ancestry. Across the four reading‐/language‐related traits included in our study, European‐ancestry sample sizes ranged from 12,411 to 27,180 participants, corresponding to approximately 80.0%–93.3% of the total GenLang samples, depending on the trait. Thus, the majority of GenLang participants were broadly aligned with the European ancestry of the ADHD GWAS, although the full GenLang summary statistics were not exclusively European. Summary statistics were accessed from the PGC portal (https://pgc.unc.edu/for‐researchers/download‐results) and the GWAS Catalog (https://www.ebi.ac.uk/gwas/).

### Heritability and genetic correlation analysis

We estimated SNP‐based heritability (h^2^_SNP) and genome‐wide genetic correlations (rg) among ADHD and reading‐/language‐related traits using linkage disequilibrium score regression (LDSC; v1.0.1) (Bulik‐Sullivan et al., 2015a, 2015b). Analyses used 1000 Genomes Phase 3 European LD scores restricted to HapMap3 SNPs (Bulik‐Sullivan, Loh, et al., 2015; Okamura et al., 2016). Univariate LDSC estimated h^2^_SNP for each trait, followed by bivariate LDSC to compute genetic correlations. The LDSC intercept was left unconstrained in the primary analyses. As a sensitivity analysis, we repeated LDSC with the univariate intercept constrained to 1 and the cross‐trait intercept constrained to 0 to examine whether the direction of the genetic correlations was consistent under a more restrictive model. These constrained estimates were not treated as primary evidence because intercept constraints can introduce bias if residual confounding or sample overlap is present. Multiple testing across the four bivariate genetic correlation tests between ADHD and the four reading‐ and language‐related traits, word reading, nonword reading, spelling, and phoneme awareness, was controlled using the Benjamini‐Hochberg false discovery rate procedure. As a conservative sensitivity correction, we also applied Bonferroni correction with a significance threshold of 0.05/4 = 0.0125.

### Partitioned heritability enrichment analysis using S‐LDSC

We used stratified LD score regression (S‐LDSC) (Finucane et al., 2015) to test whether SNP‐heritability was enriched within functional annotations for each trait separately. We applied the baseline‐LD v2.2 default annotation set from ENCODE/Roadmap (e.g., coding, conserved elements, promoters, enhancers, DHSs, intronic/transcribed regions, histone marks), restricted analyses to HapMap3 SNPs with 1000 Genomes Phase 3 European LD scores, excluded the MHC region (chr6:25–34 Mb), and used LDSC regression weights. For each annotation we report enrichment (proportion of h^2^ explained divided by the proportion of SNPs), its standard error, the nominal *p*‐value, and the FDR‐adjusted *q*‐value. Multiple testing across the full set of annotation‐by‐trait tests was controlled using the FDR‐BH, with *q* < 0.05 considered significant. Annotations that were FDR‐significant in both ADHD and a given reading‐related trait were labeled as overlapping enrichments; this descriptive overlap does not infer annotation‐stratified cross‐trait covariance or partitioned genetic correlation from S‐LDSC.

### Cross‐trait association analyses (MTAG and CPASSOC)

We applied MTAG (Turley et al., 2018) and CPASSOC (SHet as primary; SHom as sensitivity) (Zhu et al., 2015, 2021) to test ADHD with each reading‐related trait. Summary statistics were harmonized as above (effect‐allele alignment; removal of palindromic/multi‐allelic SNPs; MHC excluded). For CPASSOC, the cross‐trait sample‐overlap correlation was derived from unconstrained LDSC intercepts. Independent loci were defined by PLINK v1.9 clumping against the 1000 Genomes European LD reference (*r* ^2^ < 0.20, 1‐Mb window), retaining the lowest‐p variant per locus (Purcell et al., 2007). Significance tiers were prespecified: genome‐wide significant (GWS) if *p* < 5 × 10^−8^ in either CPASSOC (SHet) or MTAG; suggestive if both MTAG and CPASSOC showed *p* < 5 × 10^−6^ with concordant effect directions. Within each ADHD‐reading/language trait pair, defined as ADHD paired separately with word reading, nonword reading, spelling, or phoneme awareness, multiple testing across CPASSOC statistics was controlled using the Benjamini–Hochberg false discovery rate procedure; *q* < 0.05 was considered significant.

### Mendelian randomization

Two‐sample MR was conducted to evaluate potential directional relationships between ADHD and the four reading‐ and language‐related skills. For each exposure, we selected independent GWS variants as instrumental variables using a threshold of *p* < 5 × 10^−8^ and stringent LD clumping (clump_kb = 10,000; clump_*r* ^2^ = 0.001). This strategy was used to satisfy the relevance assumption by retaining variants robustly associated with the exposure and to reduce redundancy among correlated variants within the same LD region. Instrument strength was assessed using F statistics, with *F* > 10 considered to indicate low risk of weak‐instrument bias.

We recognized that the independence and exclusion‐restriction assumptions cannot be fully verified in summary‐level MR, especially for highly polygenic neurodevelopmental traits. Therefore, the inverse‐variance weighted (IVW) method under a random‐effects model was used as the primary estimator, and MR‐Egger, weighted median, and weighted mode methods were applied as complementary analyses with different assumptions about invalid instruments. Directional pleiotropy was assessed using the MR‐Egger intercept test, global horizontal pleiotropy was evaluated using the MR‐PRESSO global test, and heterogeneity across instruments was examined using Cochran's Q statistic. Bidirectional MR was performed to assess whether evidence supported ADHD liability influencing reading‐/language‐related traits or the reverse direction.

## RESULTS

### Genetic correlation

We estimated SNP‐based heritability for ADHD and the four reading‐ and language‐related traits, word reading, nonword reading, spelling, and phoneme awareness, using LDSC. SNP‐based heritability was 0.09 for ADHD and ranged from 0.17 to 0.25 across the four reading‐/language‐related traits (Table 1). Primary unconstrained bivariate LDSC showed consistently negative genetic correlations between ADHD and each of the four reading‐ and language‐related traits: word reading, nonword reading, spelling, and phoneme awareness (rg range −0.28 to −0.38). All four associations remained significant after both Benjamini‐Hochberg FDR correction and Bonferroni correction across the four tests. For illustration, ADHD‐word reading showed rg = −0.353 (SE = 0.043, *p* = 1.35 × 10^−16^), and ADHD‐spelling showed rg = −0.380 (SE = 0.047, *p* = 1.04 × 10^−15^). As sensitivity analyses, constrained LDSC estimates were directionally consistent with the primary unconstrained results. Because constrained intercepts rely on stronger assumptions, these results were interpreted only as sensitivity analyses and not as primary evidence. Full statistics are reported in Table 1 and Supporting Information S1: Tables S1–S4.

**TABLE 1:** Genome‐wide genetic correlation between reading‐ and language‐related traits and ADHD using constrained and unconstrained LDSC.

Trait1	$h_{\text{trait}1}^{2}$	Trait2	$h_{\text{trait}2}^{2}$	Constrained LDSC			Unconstrained LDSC			
				$r_{g}$	$r_{g}\_SE$	*p*	$r_{g}$	$r_{g}\_SE$	*p*	*q* (FDR‐BH)
ADHD	0.09	Word reading	0.17	−0.39	0.03	3.48 × 10^−39^	−0.35	0.04	1.35 × 10^−16^	5.40×10^−16^
ADHD	0.09	Nonword reading	0.25	−0.38	0.04	5.18 × 10^−24^	−0.28	0.04	2.15 × 10^−9^	2.87 × 10^−9^
ADHD	0.09	Spelling	0.23	−0.48	0.04	2.27 × 10^−29^	−0.38	0.05	1.03 × 10^−15^	2.06 × 10^−15^
ADHD	0.09	Phoneme awareness	0.21	−0.38	0.05	3.64 × 10^−14^	−0.28	0.06	2.64 × 10^−6^	2.64 × 10^−6^

*Note*: $h_{\text{trait}1}^{2}$ and $h_{\text{trait}2}^{2}$ indicate SNP‐based heritability estimates for Trait1 and Trait2 from univariate LDSC. $r_{g}$ indicates the genome‐wide genetic correlation from bivariate LDSC; $r_{g}\_SE$ indicates the standard error of $r_{g}$. *q* values were calculated across the four unconstrained ADHD–reading/language genetic correlation tests. All four unconstrained genetic correlations remained significant after both FDR‐BH correction (*q* < 0.05) and Bonferroni correction (α=0.0125).

Abbreviations: ADHD, attention‐deficit/hyperactivity disorder; FDR‐BH, Benjamini–Hochberg false discovery rate correctionl LDSC, linkage disequilibrium score regression.

### Partitioned heritability enrichment using S‐LDSC

Using S‐LDSC with the baseline‐LD v2.2 annotation set and Benjamini–Hochberg control across all annotation‐by‐trait tests (*q* < 0.05), we observed significant heritability enrichment concentrated in conserved and allele‐frequency‐related annotations (Supporting Information S1: Tables S5–S9). The MAF‐adjusted annotations in the baseline‐LD model capture allele‐frequency‐ and LD‐related genomic properties after accounting for minor allele frequency. These categories are useful for modeling the distribution of common‐variant heritability across genomic regions, but they should not be interpreted as specific biological pathways or cell‐type annotations. For ADHD, conserved categories showed the strongest signals, for example, Genomic Evolutionary Rate Profiling. RSsup4 (enrichment = 20.91, *p* = 1.44 × 10^−6^, *q* = 1.30 × 10^−5^) and Conserved_Primate_phastCons46way (enrichment = 14.48, *p* = 7.84 × 10^−10^, *q* = 9.88 × 10^−9^). Several MAF/diversity annotations were also significant after FDR (e.g., Nucleotide_Diversity_10kb, MAFbin1, MAF_Adj_ASMC/LLD_AFR; all *q* < 0.05).

Among reading‐related traits, word reading showed FDR‐significant enrichment in Nucleotide_Diversity_10kb (*p* = 1.27 × 10^−5^, *q* = 4.00 × 10^−4^) and additional MAF‐adjusted annotations (MAF_Adj_LLD_AFR, MAF_Adj_ASMC, MAFbin1; *q* < 0.05). For spelling, only MAF_Adj_ASMC remained significant after FDR (*p* = 3.62 × 10^−4^, *q* = 0.022). Phoneme awareness showed significant depletion in Nucleotide_Diversity_10kb (enrichment = 0.68, *p* = 4.57 × 10^−4^, *q* = 0.028) and enrichment in MAF_Adj_ASMC (*p* = 0.001, *q* = 0.038). Nonword reading had no FDR‐significant annotations.

We also noted overlapping enrichments, defined as annotations that were FDR‐significant in both ADHD and a given reading‐related trait. These overlaps were primarily observed within Nucleotide_Diversity_10kb and MAF‐adjusted categories, suggesting shared frequency/LD‐related components of the common‐variant signal rather than a specific biological pathway. The overlap between ADHD and word reading included MAF_Adj_ASMC, MAF_Adj_LLD_AFR, MAFbin1, and Nucleotide_Diversity_10kb. The overlap between ADHD and spelling included MAF_Adj_ASMC. The overlap between ADHD and phoneme awareness included MAF_Adj_ASMC and Nucleotide_Diversity_10kb. No FDR‐significant overlaps were observed for nonword reading.

### Cross‐trait association signals (MTAG and CPASSOC)

After Benjamini‐Hochberg control within each ADHD–reading/language trait pair (*q* < 0.05), MTAG and CPASSOC (SHet as the primary test; sample‐overlap from LDSC; clumping with 1000 Genomes EUR, *r* ^2^ < 0.20, 1‐Mb window) identified convergent cross‐trait association signals (Supporting Information S1: Tables S10–S13). ADHD‐word reading yielded six GWS loci by CPASSOC (*p* < 5 × 10^−8^), all supported by MTAG (*p* < 5 × 10^−6^). ADHD‐nonword reading showed no GWS loci, but four loci met a dual‐method suggestive criterion (both MTAG and CPASSOC *p* < 5 × 10^−6^ with concordant directions). ADHD‐spelling and ADHD‐phoneme awareness each showed seven GWS loci, again with dual‐method support. For transparency, CPASSOC FDR‐adjusted *q* values spanned 8.8 × 10^−15^−4.4 × 10^−8^ (word reading), ≤4.8 × 10^−6^ for suggestive nonword loci, 2.1 × 10^−13^−2.7 × 10^−8^ (spelling), and 1.4 × 10^−14−^2.7 × 10^−8^ (phoneme awareness). Representative regions include signals near PTPRF/DCC (word reading), MEF2C (spelling), and LINC00461/ST3GAL3 (phoneme awareness). Recurrent lead variants (e.g., rs1343667, rs7506904, rs7613360) indicate shared cross‐trait association architecture across attention and reading/language performance. Per‐locus MTAG p, CPASSOC‐SHet p and q, LD parameters, nearest HGNC‐approved gene, and novelty versus single‐trait GWAS are reported in Supporting Information S1: Tables S10–S13. These cross‐trait associations do not, by themselves, establish pleiotropy or colocalized causality.

### Mendelian randomization

To examine whether the observed genetic correlations were consistent with potential directional relationships, we performed bidirectional two‐sample MR analyses using independent GWS variants as instrumental variables. All retained instruments showed adequate strength, with F statistics greater than 10, indicating a low risk of weak‐instrument bias.

**FIGURE 2:**
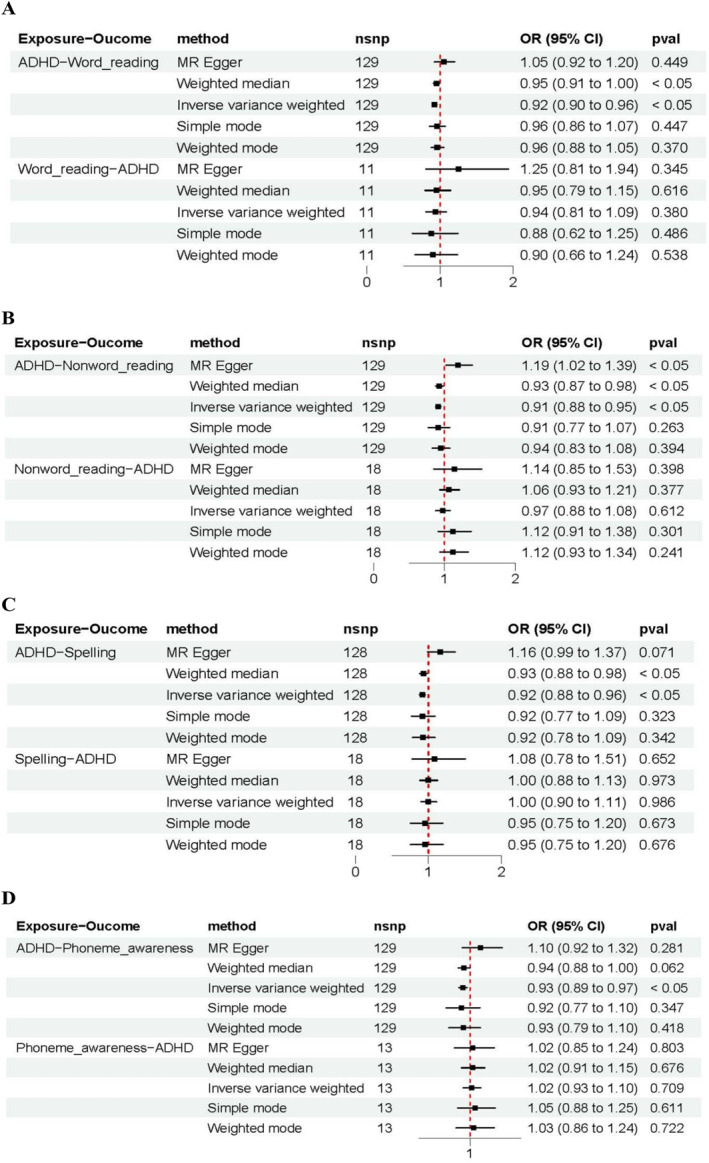
Bidirectional two‐sample Mendelian randomization (MR) analyses between ADHD and reading‐/language‐related traits. (A) ADHD and word reading; (B) ADHD and nonword reading; (C) ADHD and spelling; (D) ADHD and phoneme awareness. Estimates are shown as odds ratios with 95% confidence intervals. Exact *p*‐values are displayed for each MR method, with significant *p*‐values highlighted in bold.

In forward MR analyses, ADHD genetic liability was treated as the exposure and each reading‐/language‐related skill was treated as the outcome in separate models. The primary IVW estimates showed negative associations between ADHD genetic liability and word reading (OR = 0.92, 95% CI: 0.90–0.96, *p* = 2.12 × 10‐6), nonword reading (OR = 0.91, 95% CI: 0.88–0.95, *p* = 1.08 × 10‐5), spelling (OR = 0.92, 95% CI: 0.88–0.96, *p* = 4.46 × 10‐5), and phoneme awareness (OR = 0.93, 95% CI: 0.89–0.97, *p* = 2.12 × 10‐6) (Figure 2). However, support from complementary MR methods varied across traits. Word reading and spelling were supported by both IVW and weighted median estimates. Nonword reading showed significant IVW and weighted median estimates, although the MR‐Egger estimate differed in direction. Phoneme awareness was supported mainly by the IVW estimate and should therefore be interpreted more cautiously.

To address potential horizontal pleiotropy, we evaluated directional pleiotropy using the MR‐Egger intercept test and global horizontal pleiotropy using the MR‐PRESSO global test. No strong evidence of directional or global horizontal pleiotropy was detected. Heterogeneity tests did not indicate substantial instability across instruments. However, given the highly polygenic and neurodevelopmentally overlapping nature of ADHD and reading‐/language‐related traits, residual horizontal pleiotropy cannot be fully excluded.

In reverse MR analyses, where each reading‐/language‐related skill was treated as the exposure and ADHD as the outcome, we did not observe significant associations with ADHD risk. However, because the reading‐/language‐related GWAS had smaller sample sizes and fewer GWS instruments than the ADHD GWAS, the reverse‐direction MR analyses may have had limited power to detect modest effects. Therefore, these null findings should not be interpreted as definitive evidence against a reading‐/language‐to‐ADHD pathway. Overall, these findings are consistent with a potential directional contribution of ADHD genetic liability to poorer reading‐ and language‐related performance under standard MR assumptions. Given the modest effect sizes, limited variance explained by the instruments, potential residual pleiotropy, and variable support across complementary MR methods, these findings should be interpreted cautiously rather than as definitive evidence of causality.

## DISCUSSION

### Overview of findings

This study presents a large‐scale genome‐wide cross‐trait analysis characterizing shared genetic architecture between ADHD and key reading‐ and language‐related abilities. Using PGC and GenLang GWAS, we observed robust negative genetic correlations between ADHD and word reading, nonword reading, spelling, and phoneme awareness. Cross‐trait association analyses (MTAG and CPASSOC) identified shared loci mapping near neurodevelopmental genes (e.g., DCC, MEF2C, ST3GAL3) implicated in axon guidance, synaptic regulation, and cognitive development. Bidirectional MR findings were consistent with a potential directional contribution of ADHD genetic liability to poorer reading‐/language‐related performance, although reverse‐direction analyses may have had limited power. Together, these results support a transdiagnostic framework in which ADHD liability and reading‐/language‐related skills partly reflect overlapping polygenic influences.

Although ADHD showed significant negative genetic correlations with all four reading‐/language‐related traits, the strength and consistency of evidence varied across traits. Word reading and spelling showed relatively stronger shared signals, including larger genetic correlations and multiple GWS cross‐trait loci. In contrast, nonword reading showed a weaker genetic correlation, no GWS cross‐trait locus, and no FDR‐significant partitioned heritability enrichment, suggesting a less robust shared signal in the present analyses. Phoneme awareness showed significant genetic correlation and cross‐trait loci, but the MR evidence was supported mainly by the IVW estimate and should be interpreted cautiously. These trait‐specific differences may reflect partly distinct literacy‐related processes, including orthographic recognition, phonological decoding, spelling‐related encoding, and metalinguistic awareness, but may also be influenced by differences in GWAS sample size and statistical power.

### Relation to prior work

Our findings build upon and extend previous genetic evidence indicating that ADHD and reading‐related disorders share overlapping polygenic determinants (Ciulkinyte et al., 2025; Gialluisi et al., 2021). Prior GWAS, genetic correlation, and polygenic score studies have reported genetic overlap between ADHD, dyslexia, educational attainment, and reading‐related skills. The large dyslexia GWAS by Doust et al. identified 42 GWS loci for self‐reported dyslexia and reported a positive genetic correlation between dyslexia and ADHD (Doust et al., 2022). In contrast, we observed negative genetic correlations between ADHD and quantitative reading‐/language‐related performance. These directions are compatible because dyslexia represents impaired reading, whereas the GenLang traits reflect higher reading and language performance.

Our study complements prior work by examining quantitatively assessed component skills, word reading, nonword reading, spelling, and phoneme awareness, rather than a disorder‐level dyslexia phenotype or broad educational attainment. By combining LDSC, partitioned heritability analysis, MTAG/CPASSOC, and MR, we extended prior evidence beyond global genetic correlation and examined both shared genomic signals and potential directional relationships. Overall, our results support a broad polygenic overlap between ADHD liability and poorer reading‐/language‐related performance, while also indicating that trait‐specific differences and causal interpretations should be considered cautiously.

### Neurodevelopmental and mechanistic interpretation

Bidirectional MR findings were consistent with a potential directional contribution of ADHD genetic liability to poorer reading‐/language‐related performance, with no clear evidence supporting the reverse direction under standard MR assumptions. This aligns with neurocognitive and neuroimaging evidence showing altered activation and connectivity in left temporoparietal and inferior frontal regions that support phonological processing, verbal working memory, and reading fluency in ADHD (Demontis et al., 2023; Glasgow et al., 2020; Graham & Fisher, 2015; Langer et al., 2019; Liu et al., 2016). Such network inefficiencies can limit sustained attention and cognitive flexibility, constraining the acquisition of phonological–orthographic representations critical for literacy. The cross‐trait association and partitioned heritability results should be interpreted as complementary levels of evidence. Cross‐trait analyses identified specific loci near neurodevelopmental genes such as DCC, MEF2C, and ST3GAL3, whereas partitioned heritability analyses tested whether common‐variant heritability was enriched across broad genomic annotations. Because we did not perform formal colocalization, fine‐mapping, or SNP‐level annotation tests, individual cross‐trait loci should not be interpreted as directly explaining the annotation‐level enrichment. The overlapping MAF‐adjusted categories in S‐LDSC mainly reflect allele‐frequency‐ and LD‐related genomic properties in the baseline‐LD model, rather than specific biological pathways or cell types. Thus, these findings suggest shared features of common‐variant architecture but do not provide direct mechanistic evidence. The highlighted loci are consistent with neurodevelopmental processes such as axon guidance, synaptic regulation, and neural connectivity, many of which are active during prenatal and early postnatal brain development and continue to influence later circuit refinement relevant to attention and literacy‐related skills.

### Clinical implications and limitations

The identification of shared genetic influences between ADHD and literacy‐related skills may inform etiological models of comorbidity and support the rationale for integrated developmental assessment. However, given the modest MR effect sizes and limited variance captured by current GWAS resources, these findings should not be interpreted as supporting direct clinical genetic prediction or individualized intervention decisions. Difficulties in reading and language among children with ADHD likely reflect shared neurodevelopmental vulnerabilities rather than secondary effects of behavioral symptoms or environmental disadvantage. This insight underscores the importance of early identification of attentional difficulties as a risk marker for later literacy problems. Integrative screening approaches that assess both attentional and linguistic development could enable more effective early interventions. From a therapeutic perspective, interventions targeting domain‐general functions, such as sustained attention, working memory, and executive control, alongside structured reading and language instruction may produce greater and more durable improvements than domain‐specific training alone.

Despite these strengths, several limitations should be acknowledged. First, ancestry differences between the ADHD and GenLang GWAS should be considered. The ADHD GWAS was based on European‐ancestry participants, whereas the GenLang summary statistics used here were derived from the full meta‐analysis of reading‐ and language‐related traits. This may introduce residual ancestry‐related heterogeneity and should be addressed in future ancestry‐matched analyses. Second, the GWAS datasets used in this study capture only a limited proportion of the genetic variance underlying ADHD and reading‐/language‐related skills. Although the PGC ADHD GWAS and GenLang GWAS are among the most relevant currently available resources for the present research question, their SNP‐based heritability estimates reflect mainly common genotyped or well‐imputed variants and are lower than twin‐based heritability estimates. Rare variants, structural variants, gene–gene interactions, gene‐environment interplay, and developmental epigenetic mechanisms are not captured by these summary statistics.

Third, the reading‐/language‐related GWAS, particularly for nonword reading and phoneme awareness, had relatively modest sample sizes compared with large GWAS of broader educational or cognitive phenotypes. This may have limited power for locus discovery, cross‐trait association analyses, MR analyses, and partitioned heritability enrichment estimates. The smaller sample sizes also limited the number of robust instruments available for reverse MR analyses, reducing power to detect modest reading‐/language‐to‐ADHD effects. Conversely, although the ADHD‐to‐reading/language MR estimates were statistically significant, their effect sizes were small and the variance explained by the instruments was limited. Therefore, the MR findings should be interpreted cautiously as evidence for a modest genetic liability relationship rather than a strong causal pathway. We selected the GenLang GWAS because its phenotypes are proximal, quantitatively assessed reading‐ and language‐related skills, including word reading, nonword reading, spelling, and phoneme awareness. Dyslexia GWAS and educational‐attainment GWAS are valuable complementary resources and may provide greater predictive power because of larger sample sizes or stronger aggregate genetic signal. However, they address different levels of phenotype definition. Dyslexia GWAS captures a disorder‐level phenotype that may vary across diagnostic criteria, ascertainment strategies, languages, and educational contexts. Educational attainment is a broad distal phenotype influenced not only by reading and language ability but also by general cognitive ability, socioeconomic background, schooling opportunity, family environment, motivation, and behavioral traits. Therefore, the GenLang traits were better aligned with our objective of examining ADHD in relation to core reading‐ and language‐related skills. Future studies should triangulate findings across reading‐skill GWAS, dyslexia GWAS, and educational‐attainment GWAS to evaluate phenotype specificity.

## CONCLUSION

This study provides genome‐wide evidence for shared genetic architecture between ADHD and reading‐/language‐related abilities. Cross‐trait association signals at neurodevelopmental loci, together with enrichment in selected genomic annotations, suggest molecular convergence across attentional and literacy‐related domains. Bidirectional MR findings were consistent with a potential directional contribution of ADHD genetic liability to poorer reading‐/language‐related performance, with no clear evidence supporting the reverse direction under standard MR assumptions. However, the modest MR effect sizes, limited variance explained by current GWAS datasets, and potential residual pleiotropy indicate that causal and clinical interpretations should remain cautious. Future work incorporating larger ancestry‐matched or multi‐ancestry datasets, formal colocalization, and longitudinal designs will be essential to refine mechanistic inference and translational relevance.

## AUTHOR CONTRIBUTIONS

**Jinzhu Zhao**: Writing—original draft; software; funding acquisition; conceptualization; methodology. **Shi Huang:** Writing—review and editing. **Wei Liu:** Supervision. **Feng Wang:** Formal analysis. **Hong Qian:** Software; writing—review and editing. **Xiaolin Hu:** Writing—review and editing; funding acquisition; project administration.

## CONFLICT OF INTEREST STATEMENT

The authors declare no conflicts of interest.

## ETHICAL CONSIDERATIONS

Not applicable. This study used publicly available summary‐level data and did not involve collection of new data from human participants.

## Supporting information

Supporting Information S1

## Data Availability

The data that support the findings of this study are available in Psychiatric Genomics Consortium (PGC) download portal at https://pgc.unc.edu/for‐researchers/download‐results. These data were derived from the following resources available in the public domain: ‐ PGC ADHD 2022 summary statistics (Demontis et al., 2023) European, https://pgc.unc.edu/for‐researchers/download‐results ‐ GWAS Catalog—GenLang Consortium reading/language traits, https://www.ebi.ac.uk/gwas/.
