# Supplementary material for: Shared genetics between ADHD and reading/language abilities: Genome‐wide correlations, stratified enrichment, cross‐trait association, and mendelian randomization
Source: JCPP Adv. 2026 Jul 18:e70148. Online ahead of print. doi: 10.1002/jcv2.70148 (PMC13379746; doi:10.1002/jcv2.70148)
Supplement: Supplementary file 1 — Supporting Information S1 [file JCV2-9999-e70148-s001.docx]

**Shared genetics between ADHD and reading/language abilities: genome-wide correlations, stratified enrichment, cross-trait association, and Mendelian randomization**

**Supporting Information**

**Table S1. Genetic correlations estimated using LDSC with and without constrained intercept between ADHD and word reading**

**LDSC test unconstrained intercept**

Heritability of ADHD

---------------------------

Total Observed scale h2: 0.0946 (0.0045)

Lambda GC: 1.3546

Mean Chi^2: 1.4487

Intercept: 1.0268 (0.0095)

Ratio: 0.0596 (0.0213)

Heritability of word reading

-----------------------------

Total Observed scale h2: 0.1731 (0.0169)

Lambda GC: 1.0864

Mean Chi^2: 1.1022

Intercept: 0.9851 (0.0075)

Ratio < 0 (usually indicates GC correction).

Genetic Covariance

------------------

Total Observed scale gencov: -0.0451 (0.0053)

Mean z1*z2: -0.0837

Intercept: -0.0059 (0.0059)

Genetic Correlation

-------------------

Genetic Correlation: -0.3527 (0.0427)

Z-score: -8.2691

P: 1.3502e-16

**LDSC test constrained intercept**

Heritability of ADHD

---------------------------

Total Observed scale h2: 0.1023 (0.0032)

Lambda GC: 1.3546

Mean Chi^2: 1.4487

Intercept: constrained to 1

Heritability of word reading

-----------------------------

Total Observed scale h2: 0.1491 (0.0113)

Lambda GC: 1.0864

Mean Chi^2: 1.1022

Intercept: constrained to 1

Genetic Covariance

------------------

Total Observed scale gencov: -0.0492 (0.0035)

Mean z1*z2: -0.0837

Intercept: constrained to 0

Genetic Correlation

-------------------

Genetic Correlation: -0.3981 (0.0304)

Z-score: -13.0956

P: 3.4877e-39

**Table S2. Genetic correlations estimated using LDSC with and without constrained intercept between ADHD and nonword reading**

**LDSC test unconstrained intercept**

Heritability of ADHD

---------------------------

Total Observed scale h2: 0.0942 (0.0045)

Lambda GC: 1.3546

Mean Chi^2: 1.4491

Intercept: 1.0281 (0.0094)

Ratio: 0.0626 (0.021)

Heritability of nonword reading

-----------------------------

Total Observed scale h2: 0.0894 (0.0297)

Lambda GC: 0.8753

Mean Chi^2: 0.8995

Intercept: 0.8744 (0.0061)

Ratio: NA (mean chi^2 < 1)

Genetic Covariance

------------------

Total Observed scale gencov: -0.022 (0.0075)

Mean z1*z2: -0.0265

Intercept: -0.0026 (0.0053)

Genetic Correlation

-------------------

Genetic Correlation: -0.2395 (0.094)

Z-score: -2.5469

P: 0.0109

**LDSC test constrained intercept**

Heritability of ADHD

---------------------------

Total Observed scale h2: 0.1023 (0.0032)

Lambda GC: 1.3546

Mean Chi^2: 1.4488

Intercept: constrained to 1

Heritability of nonword reading

-----------------------------

Total Observed scale h2: 0.1832 (0.0189)

Lambda GC: 1.0649

Mean Chi^2: 1.0675

Intercept: constrained to 1

Genetic Covariance

------------------

Total Observed scale gencov: -0.0517 (0.0046)

Mean z1*z2: -0.0637

Intercept: constrained to 0

Genetic Correlation

-------------------

Genetic Correlation: -0.3779 (0.0374)

Z-score: -10.1062

P: 5.1846e-24

**Table S3. Genetic correlations estimated using LDSC with and without constrained intercept between ADHD and spelling**

**LDSC test unconstrained intercept**

Heritability of ADHD

---------------------------

Total Observed scale h2: 0.0944 (0.0045)

Lambda GC: 1.3546

Mean Chi^2: 1.4488

Intercept: 1.0274 (0.0095)

Ratio: 0.061 (0.0212)

Heritability of spelling

-----------------------------

Total Observed scale h2: 0.2342 (0.0298)

Lambda GC: 1.0527

Mean Chi^2: 1.0621

Intercept: 0.9738 (0.0068)

Ratio < 0 (usually indicates GC correction).

Genetic Covariance

------------------

Total Observed scale gencov: -0.0565 (0.0073)

Mean z1*z2: -0.0793

Intercept: -0.0051 (0.0055)

Genetic Correlation

-------------------

Genetic Correlation: -0.3802 (0.0474)

Z-score: -8.0226

P: 1.035e-15

**LDSC test constrained intercept**

Heritability of ADHD

---------------------------

Total Observed scale h2: 0.1023 (0.0032)

Lambda GC: 1.3546

Mean Chi^2: 1.4488

Intercept: constrained to 1

Heritability of spelling

-----------------------------

Total Observed scale h2: 0.1601 (0.0202)

Lambda GC: 1.0527

Mean Chi^2: 1.0621

Intercept: constrained to 1

Genetic Covariance

------------------

Total Observed scale gencov: -0.0612 (0.0049)

Mean z1*z2: -0.0793

Intercept: constrained to 0

Genetic Correlation

-------------------

Genetic Correlation: -0.4781 (0.0425)

Z-score: -11.2518

P: 2.2692e-29

**Table S5. Partitioned SNP heritability in ADHD**

| **Category** | **Prop._SNPs** | **Prop._h2** | **Prop._h2_std_error** | **Enrichment** | **Enrichment_std_error** | **Enrichment_p** | **q (FDR-BH)** |
| --- | --- | --- | --- | --- | --- | --- | --- |
| Coding_UCSC | 0.01425914 | 0.043103101 | 0.021118995 | 3.022840188 | 1.481084807 | 0.173368416 | 0.377 |
| **Conserved_LindbladToh** | 0.024670538 | 0.21370243 | 0.04065232 | 8.662252507 | 1.647808406 | 6.46E-06 | 5.09e-05 |
| CTCF_Hoffman | 0.023814664 | 0.021714337 | 0.034108727 | 0.911805309 | 1.432257334 | 0.950899903 | 0.966 |
| DGF_ENCODE | 0.135362603 | 0.292611188 | 0.086628472 | 2.161684105 | 0.639973449 | 0.070169039 | 0.184 |
| DHS_peaks_Trynka | 0.109959825 | 0.087131479 | 0.077345922 | 0.792393758 | 0.70340165 | 0.76762009 | 0.864 |
| DHS_Trynka | 0.165742769 | 0.214635013 | 0.08248582 | 1.294988699 | 0.497673714 | 0.554230946 | 0.743 |
| Enhancer_Andersson | 0.004313758 | 0.025714476 | 0.016073141 | 5.961037607 | 3.726017795 | 0.181090328 | 0.38 |
| Enhancer_Hoffman | 0.041923223 | 0.04760417 | 0.03254337 | 1.135508348 | 0.77626115 | 0.861545777 | 0.926 |
| **FetalDHS_Trynka** | 0.083682888 | 0.244571251 | 0.063234539 | 2.922595731 | 0.755644796 | 0.011647124 | 0.0439 |
| H3K27ac_Hnisz | 0.389043808 | 0.415522225 | 0.032407325 | 1.068060245 | 0.083299938 | 0.419741003 | 0.639 |
| H3K27ac_PGC2 | 0.268521272 | 0.301349724 | 0.04656093 | 1.122256429 | 0.173397546 | 0.482282074 | 0.691 |
| H3K4me1_peaks_Trynka | 0.168870181 | 0.271844535 | 0.064116271 | 1.609784116 | 0.379677869 | 0.107615028 | 0.261 |
| **H3K4me1_Trynka** | 0.42345507 | 0.588609151 | 0.0639231 | 1.390015595 | 0.150956039 | 0.011858397 | 0.0439 |
| H3K4me3_peaks_Trynka | 0.041368465 | 0.029295003 | 0.039083059 | 0.708148166 | 0.944754871 | 0.756753633 | 0.864 |
| H3K4me3_Trynka | 0.132886742 | 0.184587398 | 0.044348438 | 1.389058043 | 0.333731096 | 0.244220335 | 0.453 |
| **H3K9ac_peaks_Trynka** | 0.038186366 | 0.157282173 | 0.047419764 | 4.118804399 | 1.241798287 | 0.015348426 | 0.0509 |
| H3K9ac_Trynka | 0.125302311 | 0.10521921 | 0.048691502 | 0.83972282 | 0.388592214 | 0.678121391 | 0.822 |
| Intron_UCSC | 0.387451333 | 0.416874364 | 0.024532017 | 1.075939942 | 0.063316384 | 0.228014006 | 0.435 |
| PromoterFlanking_Hoffman | 0.008276914 | -0.003243631 | 0.022973708 | -0.391888957 | 2.775636934 | 0.616572124 | 0.777 |
| Promoter_UCSC | 0.046335285 | 0.066874951 | 0.030404912 | 1.443283484 | 0.656193493 | 0.49969726 | 0.7 |
| Repressed_Hoffman | 0.460018094 | 0.426172139 | 0.065167964 | 0.926424732 | 0.141663915 | 0.604883467 | 0.777 |
| SuperEnhancer_Hnisz | 0.167206746 | 0.198887024 | 0.017777775 | 1.1894677 | 0.106322115 | 0.078897768 | 0.199 |
| TFBS_ENCODE | 0.130971511 | 0.151086107 | 0.063347697 | 1.153579936 | 0.483675394 | 0.751127113 | 0.864 |
| Transcr_Hoffman | 0.345192604 | 0.317167524 | 0.06034407 | 0.918813207 | 0.174812754 | 0.642303901 | 0.793 |
| TSS_Hoffman | 0.01780694 | 0.050457726 | 0.025694879 | 2.833598941 | 1.44296994 | 0.206595961 | 0.42 |
| UTR_3_UCSC | 0.01116813 | 0.045851314 | 0.019075384 | 4.105549747 | 1.708019458 | 0.06948664 | 0.184 |
| UTR_5_UCSC | 0.00546018 | -0.002068186 | 0.012261757 | -0.378776163 | 2.245669013 | 0.538781371 | 0.738 |
| WeakEnhancer_Hoffman | 0.020905666 | 0.054771039 | 0.032210047 | 2.619913615 | 1.540732874 | 0.297732985 | 0.507 |
| **GERP.NS** | 1.745316256 | 3.313311934 | 0.17325456 | 1.898402036 | 0.09926829 | 8.10E-16 | 5.1e-14 |
| **GERP.RSsup4** | 0.008149925 | 0.170386321 | 0.033063909 | 20.90648886 | 4.056958619 | 1.44E-06 | 1.3e-05 |
| **MAFbin1** | 0.102357276 | 0.050149072 | 0.014527352 | 0.489941445 | 0.141927886 | 0.000157498 | 0.000992 |
| **MAFbin2** | 0.099855414 | 0.038617883 | 0.014812286 | 0.386737996 | 0.148337331 | 2.43E-05 | 0.00017 |
| MAFbin3 | 0.099606637 | 0.097846787 | 0.015439072 | 0.982332005 | 0.155000436 | 0.90941609 | 0.939 |
| **MAFbin4** | 0.100695687 | 0.062600658 | 0.016661131 | 0.621681625 | 0.165460225 | 0.022312962 | 0.0703 |
| MAFbin5 | 0.098317123 | 0.105988842 | 0.02106916 | 1.078030352 | 0.214297971 | 0.715155083 | 0.85 |
| MAFbin6 | 0.0996422 | 0.096293204 | 0.022566292 | 0.966389779 | 0.226473238 | 0.881835652 | 0.926 |
| MAFbin7 | 0.099733793 | 0.129652986 | 0.02147097 | 1.299990519 | 0.215282794 | 0.164063631 | 0.369 |
| **MAFbin8** | 0.100229335 | 0.162320494 | 0.024322308 | 1.619490885 | 0.242666563 | 0.010247218 | 0.043 |
| **MAFbin9** | 0.101099971 | 0.142493097 | 0.020766656 | 1.409427679 | 0.205407139 | 0.044324435 | 0.127 |
| MAFbin10 | 0.098462564 | 0.114036977 | 0.018057787 | 1.158175982 | 0.183397488 | 0.387252199 | 0.626 |
| **MAF_Adj_Predicted_Allele_Age** | 3.39E-06 | -0.400010262 | 0.072141074 | -117912.3482 | 21265.26318 | 7.81E-07 | 8.2e-06 |
| **MAF_Adj_LLD_AFR** | 0.002796474 | -0.347879418 | 0.040505439 | -124.3992931 | 14.48446707 | 1.55E-10 | 2.44e-09 |
| Recomb_Rate_10kb | 1.552424442 | 1.510878264 | 0.177757205 | 0.973237874 | 0.114502967 | 0.814031481 | 0.9 |
| **Nucleotide_Diversity_10kb** | 4.608695341 | 3.742299456 | 0.116392464 | 0.812008427 | 0.02525497 | 7.10E-12 | 1.49e-10 |
| **Backgrd_Selection_Stat** | 0.177719812 | 0.194254671 | 0.006906661 | 1.093038916 | 0.038862641 | 0.013470436 | 0.0471 |
| **CpG_Content_50kb** | 0.010051874 | 0.010762394 | 0.000203103 | 1.07068532 | 0.020205439 | 0.000960304 | 0.0055 |
| **MAF_Adj_ASMC** | -2.35E-14 | -0.4394332 | 0.059832442 | 1.8711E+13 | -2.54766E+12 | 9.84E-13 | 3.1e-11 |
| GTEx_eQTL_MaxCPP | 0.010340409 | 0.030503429 | 0.013764951 | 2.949924685 | 1.331180481 | 0.143484372 | 0.335 |
| BLUEPRINT_H3K27acQTL_MaxCPP | 0.016545549 | 0.033843453 | 0.013829225 | 2.045471738 | 0.835827507 | 0.21812893 | 0.429 |
| BLUEPRINT_H3K4me1QTL_MaxCPP | 0.013372536 | 0.004190312 | 0.011459267 | 0.313352125 | 0.856925548 | 0.426063976 | 0.639 |
| BLUEPRINT_DNA_methylation_MaxCPP | 0.031730971 | 0.046841234 | 0.020943132 | 1.476199186 | 0.660021758 | 0.469944905 | 0.689 |
| synonymous | 0.003120031 | 0.002510092 | 0.017353471 | 0.804508797 | 5.561954995 | 0.971912275 | 0.972 |
| non_synonymous | 0.002715412 | 0.000817425 | 0.011489927 | 0.301031777 | 4.231375924 | 0.868513148 | 0.926 |
| **Conserved_Vertebrate_phastCons46way** | 0.029436054 | 0.132773706 | 0.03869443 | 4.510580957 | 1.314525009 | 0.008427409 | 0.0379 |
| **Conserved_Mammal_phastCons46way** | 0.021439455 | 0.151800715 | 0.040572659 | 7.080437201 | 1.892429596 | 0.001388298 | 0.00729 |
| **Conserved_Primate_phastCons46way** | 0.019269072 | 0.278990201 | 0.03976253 | 14.47865291 | 2.063541542 | 7.84E-10 | 9.88e-09 |
| **BivFlnk** | 0.013549379 | 0.079700175 | 0.022119272 | 5.882201478 | 1.632493454 | 0.002982467 | 0.0145 |
| Human_Promoter_Villar | 0.015217007 | 0.034339704 | 0.019219645 | 2.25666607 | 1.263037139 | 0.319940532 | 0.53 |
| Human_Enhancer_Villar | 0.033227431 | 0.023134916 | 0.01957506 | 0.696259533 | 0.589123492 | 0.604456429 | 0.777 |
| Ancient_Sequence_Age_Human_Promoter | 0.004170665 | 0.022841379 | 0.016417338 | 5.476674998 | 3.936383313 | 0.253921163 | 0.457 |
| Ancient_Sequence_Age_Human_Enhancer | 0.005138095 | 0.021800358 | 0.015726012 | 4.242887564 | 3.060669975 | 0.294560063 | 0.507 |
| Human_Enhancer_Villar_Species_Enhancer_Count | 0.066283084 | 0.10449335 | 0.045941108 | 1.576470862 | 0.693104563 | 0.408110247 | 0.639 |
| **Human_Promoter_Villar_ExAC** | 0.002496662 | 0.027781365 | 0.011431757 | 11.1274026 | 4.57881631 | 0.026393981 | 0.0792 |

Note: Bold values indicate annotations surviving Benjamini-Hochberg FDR correction at *q*<0.05.

**Table S6. Partitioned SNP heritability in Word reading**

| Category | Prop._SNPs | Prop._h2 | Prop._h2_std_error | Enrichment | Enrichment_std_error | Enrichment_p | q (FDR-BH) |
| --- | --- | --- | --- | --- | --- | --- | --- |
| Coding_UCSC | 0.01425914 | -0.01384921 | 0.047597871 | -0.97125129 | 3.338060483 | 0.555632572 | 0.761 |
| Conserved_LindbladToh | 0.024670538 | 0.099779029 | 0.079542388 | 4.044461007 | 3.224185391 | 0.344535524 | 0.692 |
| CTCF_Hoffman | 0.023814664 | -0.00541245 | 0.078073383 | -0.2272737 | 3.278374313 | 0.70903178 | 0.83 |
| DGF_ENCODE | 0.135362603 | -0.00079976 | 0.196823369 | -0.00590828 | 1.454045386 | 0.486699667 | 0.761 |
| DHS_peaks_Trynka | 0.109959825 | 0.199131727 | 0.167959033 | 1.810949838 | 1.527458166 | 0.594459256 | 0.797 |
| DHS_Trynka | 0.165742769 | 0.090622313 | 0.190523183 | 0.546764808 | 1.149511282 | 0.693133615 | 0.83 |
| Enhancer_Andersson | 0.004313758 | -0.01045697 | 0.035384374 | -2.42409711 | 8.202678467 | 0.676190007 | 0.83 |
| Enhancer_Hoffman | 0.041923223 | -0.00951537 | 0.077657081 | -0.22697143 | 1.852364276 | 0.50450776 | 0.761 |
| FetalDHS_Trynka | 0.083682888 | 0.044476147 | 0.141827197 | 0.531484374 | 1.694817195 | 0.782232796 | 0.874 |
| H3K27ac_Hnisz | 0.389043808 | 0.438457327 | 0.069110777 | 1.127012737 | 0.177642661 | 0.480683675 | 0.761 |
| H3K27ac_PGC2 | 0.268521272 | 0.214860655 | 0.106096987 | 0.800162508 | 0.395115761 | 0.612748903 | 0.804 |
| H3K4me1_peaks_Trynka | 0.168870181 | 0.262995265 | 0.149223492 | 1.557381317 | 0.88365803 | 0.527177608 | 0.761 |
| H3K4me1_Trynka | 0.42345507 | 0.504474317 | 0.136447418 | 1.191329029 | 0.322224075 | 0.554291456 | 0.761 |
| H3K4me3_peaks_Trynka | 0.041368465 | 0.038795726 | 0.088657315 | 0.937809175 | 2.143113468 | 0.976832402 | 0.981 |
| H3K4me3_Trynka | 0.132886742 | 0.168325682 | 0.092839295 | 1.266685292 | 0.69863474 | 0.703923856 | 0.83 |
| H3K9ac_peaks_Trynka | 0.038186366 | 0.168535971 | 0.10458491 | 4.41351164 | 2.738802368 | 0.215266531 | 0.612 |
| H3K9ac_Trynka | 0.125302311 | 0.035884039 | 0.106865021 | 0.286379708 | 0.852857542 | 0.399578281 | 0.699 |
| Intron_UCSC | 0.387451333 | 0.440377779 | 0.056502703 | 1.136601534 | 0.145831743 | 0.348252811 | 0.692 |
| PromoterFlanking_Hoffman | 0.008276914 | 0.049113647 | 0.047333215 | 5.933811519 | 5.718703318 | 0.386491723 | 0.699 |
| Promoter_UCSC | 0.046335285 | 0.060339953 | 0.067862759 | 1.302246291 | 1.464602167 | 0.836726541 | 0.906 |
| Repressed_Hoffman | 0.460018094 | 0.653366949 | 0.129970852 | 1.420307067 | 0.282534217 | 0.133067797 | 0.493 |
| SuperEnhancer_Hnisz | 0.167206746 | 0.229690324 | 0.036201977 | 1.373690533 | 0.216510262 | 0.089756445 | 0.435 |
| TFBS_ENCODE | 0.130971511 | 0.167503204 | 0.137488586 | 1.278928547 | 1.049759484 | 0.790524791 | 0.874 |
| Transcr_Hoffman | 0.345192604 | 0.206250141 | 0.114969281 | 0.597492932 | 0.333058355 | 0.226957769 | 0.612 |
| **TSS_Hoffman** | 0.01780694 | 0.13398871 | 0.054120488 | 7.524521934 | 3.03929189 | 0.034188044 | 0.239 |
| UTR_3_UCSC | 0.01116813 | -0.01974619 | 0.033842775 | -1.76808403 | 3.030299069 | 0.356801008 | 0.692 |
| UTR_5_UCSC | 0.00546018 | -0.01230095 | 0.029319277 | -2.25284682 | 5.369654052 | 0.545604782 | 0.761 |
| WeakEnhancer_Hoffman | 0.020905666 | 0.052453194 | 0.073100786 | 2.50904196 | 3.496697274 | 0.667663779 | 0.83 |
| GERP.NS | 1.745316256 | 2.19410904 | 0.300530727 | 1.257141238 | 0.172192705 | 0.131858611 | 0.493 |
| GERP.RSsup4 | 0.008149925 | 0.067367253 | 0.048480242 | 8.265996528 | 5.948550484 | 0.217028208 | 0.612 |
| **MAFbin1** | 0.102357276 | 0.007524453 | 0.032180679 | 0.073511656 | 0.314395618 | 0.002027202 | 0.0319 |
| MAFbin2 | 0.099855414 | 0.054493195 | 0.032966895 | 0.545720986 | 0.330146296 | 0.160334024 | 0.532 |
| MAFbin3 | 0.099606637 | 0.043469207 | 0.034769299 | 0.436408738 | 0.349066086 | 0.104381919 | 0.47 |
| MAFbin4 | 0.100695687 | 0.123638182 | 0.036942574 | 1.227839897 | 0.366873449 | 0.535123385 | 0.761 |
| MAFbin5 | 0.098317123 | 0.105883447 | 0.040479923 | 1.076958358 | 0.411728112 | 0.851842641 | 0.906 |
| MAFbin6 | 0.0996422 | 0.137353617 | 0.035617337 | 1.378468325 | 0.357452332 | 0.289243307 | 0.675 |
| MAFbin7 | 0.099733793 | 0.158614396 | 0.050391399 | 1.590377646 | 0.505259021 | 0.242139713 | 0.612 |
| MAFbin8 | 0.100229335 | 0.149810391 | 0.037960184 | 1.494676105 | 0.378733271 | 0.183105901 | 0.577 |
| MAFbin9 | 0.101099971 | 0.090194396 | 0.035679501 | 0.892130785 | 0.352913068 | 0.76022435 | 0.871 |
| MAFbin10 | 0.098462564 | 0.129018716 | 0.028872665 | 1.31033269 | 0.293234948 | 0.289013037 | 0.675 |
| MAF_Adj_Predicted_Allele_Age | 3.39E-06 | -0.02747916 | 0.157050405 | -8100.1239 | 46294.26731 | 0.862655328 | 0.906 |
| **MAF_Adj_LLD_AFR** | 0.002796474 | -0.42823036 | 0.067918601 | -153.132238 | 24.28722569 | 1.14E-05 | 0.0004 |
| Recomb_Rate_10kb | 1.552424442 | 2.134331782 | 0.351112052 | 1.374837785 | 0.226170139 | 0.124221108 | 0.493 |
| **Nucleotide_Diversity_10kb** | 4.608695341 | 3.717144519 | 0.212709304 | 0.80655028 | 0.046153909 | 1.27E-05 | 0.0004 |
| Backgrd_Selection_Stat | 0.177719812 | 0.198858224 | 0.012462893 | 1.118942353 | 0.07012664 | 0.067662295 | 0.426 |
| **CpG_Content_50kb** | 0.010051874 | 0.010911755 | 0.000345457 | 1.085544425 | 0.034367403 | 0.022393838 | 0.176 |
| **MAF_Adj_ASMC** | -2.35E-14 | -0.32989667 | 0.100621787 | 1.4047E+13 | -4.28446E+12 | 0.000864357 | 0.0182 |
| GTEx_eQTL_MaxCPP | 0.010340409 | 0.043231036 | 0.034646052 | 4.18078567 | 3.350549336 | 0.336209342 | 0.692 |
| **BLUEPRINT_H3K27acQTL_MaxCPP** | 0.016545549 | 0.089894062 | 0.026298302 | 5.433126534 | 1.58944873 | 0.00627053 | 0.079 |
| BLUEPRINT_H3K4me1QTL_MaxCPP | 0.013372536 | 0.059458732 | 0.026296714 | 4.446331977 | 1.966471765 | 0.08199582 | 0.43 |
| BLUEPRINT_DNA_methylation_MaxCPP | 0.031730971 | 0.092004036 | 0.039797743 | 2.899502646 | 1.254223891 | 0.144301206 | 0.505 |
| synonymous | 0.003120031 | -0.03291703 | 0.030774975 | -10.5502261 | 9.863676587 | 0.242820771 | 0.612 |
| non_synonymous | 0.002715412 | 0.003308486 | 0.024562952 | 1.218410495 | 9.045756508 | 0.980769442 | 0.981 |
| Conserved_Vertebrate_phastCons46way | 0.029436054 | 0.08779672 | 0.079528953 | 2.982625295 | 2.701753159 | 0.464114276 | 0.761 |
| Conserved_Mammal_phastCons46way | 0.021439455 | 0.089957751 | 0.080314381 | 4.195897299 | 3.746101818 | 0.394508032 | 0.699 |
| Conserved_Primate_phastCons46way | 0.019269072 | 0.155373822 | 0.078382902 | 8.063378706 | 4.067808961 | 0.080958355 | 0.43 |
| BivFlnk | 0.013549379 | 0.041181364 | 0.044856756 | 3.039354413 | 3.31061354 | 0.539988146 | 0.761 |
| **Human_Promoter_Villar** | 0.015217007 | 0.118865677 | 0.042957465 | 7.811370205 | 2.822990368 | 0.016200769 | 0.162 |
| Human_Enhancer_Villar | 0.033227431 | 0.017120767 | 0.043701176 | 0.515260015 | 1.315213806 | 0.71131302 | 0.83 |
| Ancient_Sequence_Age_Human_Promoter | 0.004170665 | 0.005761607 | 0.032303516 | 1.381459756 | 7.745410435 | 0.960742459 | 0.981 |
| Ancient_Sequence_Age_Human_Enhancer | 0.005138095 | 0.037931481 | 0.034192598 | 7.382401981 | 6.654723024 | 0.339806036 | 0.692 |
| Human_Enhancer_Villar_Species_Enhancer_Count | 0.066283084 | -0.02748404 | 0.104279743 | -0.41464643 | 1.573248221 | 0.362564822 | 0.692 |
| **Human_Promoter_Villar_ExAC** | 0.002496662 | 0.049819948 | 0.020309612 | 19.95462166 | 8.134705814 | 0.018029072 | 0.162 |

Note: Bold values indicate annotations surviving Benjamini-Hochberg FDR correction at *q*<0.05.

**Table S7. Partitioned SNP heritability in Spelling**

| Category | Prop._SNPs | Prop._h2 | Prop._h2_std_error | Enrichment | Enrichment_std_error | Enrichment_p | q (FDR-BH) |
| --- | --- | --- | --- | --- | --- | --- | --- |
| Coding_UCSC | 0.01425914 | -0.078721921 | 0.072356426 | -5.520804284 | 5.074389253 | 0.190524174 | 0.585 |
| Conserved_LindbladToh | 0.024670538 | 0.291871157 | 0.14343746 | 11.83075765 | 5.814119642 | 0.050412135 | 0.358 |
| CTCF_Hoffman | 0.023814664 | -0.12936356 | 0.119356057 | -5.432096739 | 5.01187233 | 0.182687143 | 0.585 |
| DGF_ENCODE | 0.135362603 | -0.34687764 | 0.304583978 | -2.562581034 | 2.250133867 | 0.098091933 | 0.45 |
| DHS_peaks_Trynka | 0.109959825 | -0.062059469 | 0.272039278 | -0.564383117 | 2.473987916 | 0.518499363 | 0.695 |
| DHS_Trynka | 0.165742769 | -0.204045387 | 0.323230382 | -1.231096763 | 1.950192964 | 0.236467077 | 0.585 |
| Enhancer_Andersson | 0.004313758 | 0.050638021 | 0.059858234 | 11.73872432 | 13.87612105 | 0.441667251 | 0.635 |
| Enhancer_Hoffman | 0.041923223 | -0.096210096 | 0.125424064 | -2.294911717 | 2.991756223 | 0.267981092 | 0.585 |
| FetalDHS_Trynka | 0.083682888 | 0.209962362 | 0.247852013 | 2.509023856 | 2.961800426 | 0.612443567 | 0.728 |
| H3K27ac_Hnisz | 0.389043808 | 0.26087993 | 0.123453523 | 0.670566976 | 0.317325506 | 0.279439333 | 0.585 |
| H3K27ac_PGC2 | 0.268521272 | 0.074728676 | 0.17603739 | 0.278297041 | 0.655580798 | 0.25452002 | 0.585 |
| H3K4me1_peaks_Trynka | 0.168870181 | 0.348696725 | 0.290427348 | 2.06488039 | 1.719826118 | 0.536088051 | 0.7 |
| H3K4me1_Trynka | 0.42345507 | 0.718579145 | 0.250621971 | 1.696943068 | 0.591850209 | 0.237486891 | 0.585 |
| H3K4me3_peaks_Trynka | 0.041368465 | 0.232879351 | 0.161399441 | 5.629393037 | 3.901509017 | 0.219608159 | 0.585 |
| H3K4me3_Trynka | 0.132886742 | 0.011105798 | 0.160960484 | 0.083573406 | 1.211260666 | 0.443656619 | 0.635 |
| H3K9ac_peaks_Trynka | 0.038186366 | 0.04271791 | 0.163107221 | 1.118669158 | 4.27134703 | 0.977835111 | 0.978 |
| H3K9ac_Trynka | 0.125302311 | 0.054216363 | 0.177424236 | 0.432684463 | 1.415969378 | 0.68559567 | 0.8 |
| Intron_UCSC | 0.387451333 | 0.530947858 | 0.081351108 | 1.370360127 | 0.209964714 | 0.068876767 | 0.434 |
| PromoterFlanking_Hoffman | 0.008276914 | 0.026714828 | 0.079786612 | 3.227631451 | 9.6396571 | 0.817754721 | 0.873 |
| Promoter_UCSC | 0.046335285 | -0.131335078 | 0.112170256 | -2.834450656 | 2.420838826 | 0.104859199 | 0.45 |
| Repressed_Hoffman | 0.460018094 | 0.712670155 | 0.264295757 | 1.549222008 | 0.574533394 | 0.321178335 | 0.595 |
| SuperEnhancer_Hnisz | 0.167206746 | 0.153001837 | 0.059289739 | 0.915045838 | 0.354589396 | 0.809305025 | 0.873 |
| TFBS_ENCODE | 0.130971511 | 0.057707679 | 0.246467229 | 0.440612459 | 1.881838482 | 0.766983562 | 0.863 |
| Transcr_Hoffman | 0.345192604 | 0.395639119 | 0.211369965 | 1.146140195 | 0.612324722 | 0.811662472 | 0.873 |
| TSS_Hoffman | 0.01780694 | -0.008393659 | 0.08302449 | -0.471370097 | 4.662479389 | 0.753139831 | 0.863 |
| UTR_3_UCSC | 0.01116813 | -0.043815471 | 0.065412483 | -3.923259307 | 5.857066601 | 0.394439811 | 0.635 |
| UTR_5_UCSC | 0.00546018 | -0.025250212 | 0.038441206 | -4.624428628 | 7.040282026 | 0.421308753 | 0.635 |
| WeakEnhancer_Hoffman | 0.020905666 | -0.077166604 | 0.118651367 | -3.691181282 | 5.675560197 | 0.396480473 | 0.635 |
| GERP.NS | 1.745316256 | 2.726878237 | 0.518457742 | 1.562397776 | 0.297056617 | 0.051157815 | 0.358 |
| GERP.RSsup4 | 0.008149925 | 0.07711394 | 0.086260122 | 9.461919998 | 10.58416115 | 0.411121322 | 0.635 |
| MAFbin1 | 0.102357276 | 0.036934257 | 0.054493658 | 0.360836654 | 0.532386748 | 0.209463376 | 0.585 |
| **MAFbin2** | 0.099855414 | -0.014487747 | 0.061978934 | -0.145087247 | 0.620686766 | 0.035177562 | 0.317 |
| MAFbin3 | 0.099606637 | 0.098047486 | 0.050609011 | 0.984346915 | 0.508088748 | 0.975445263 | 0.978 |
| MAFbin4 | 0.100695687 | 0.067047628 | 0.056499524 | 0.665844094 | 0.561091794 | 0.544811406 | 0.7 |
| MAFbin5 | 0.098317123 | 0.031657419 | 0.061443194 | 0.32199294 | 0.624949064 | 0.270633073 | 0.585 |
| MAFbin6 | 0.0996422 | 0.067321698 | 0.055622415 | 0.675634394 | 0.558221466 | 0.562010365 | 0.705 |
| MAFbin7 | 0.099733793 | 0.160920814 | 0.062109256 | 1.613503397 | 0.622750365 | 0.313456708 | 0.595 |
| MAFbin8 | 0.100229335 | 0.035250231 | 0.057340756 | 0.35169575 | 0.572095544 | 0.259916848 | 0.585 |
| **MAFbin9** | 0.101099971 | 0.274478109 | 0.073359874 | 2.714917792 | 0.725617162 | 0.008114153 | 0.128 |
| **MAFbin10** | 0.098462564 | 0.242830104 | 0.063683025 | 2.466217557 | 0.64677399 | 0.014042731 | 0.175 |
| MAF_Adj_Predicted_Allele_Age | 3.39E-06 | 0.141483363 | 0.275810695 | 41705.51888 | 81301.63112 | 0.591149796 | 0.716 |
| MAF_Adj_LLD_AFR | 0.002796474 | -0.229582884 | 0.130029362 | -82.09726392 | 46.49760779 | 0.142563636 | 0.528 |
| Recomb_Rate_10kb | 1.552424442 | 2.273607368 | 0.610460402 | 1.464552674 | 0.393230347 | 0.287748712 | 0.585 |
| Nucleotide_Diversity_10kb | 4.608695341 | 4.059088401 | 0.358605762 | 0.880745656 | 0.077810689 | 0.107098591 | 0.45 |
| Backgrd_Selection_Stat | 0.177719812 | 0.202756155 | 0.02125831 | 1.140875366 | 0.119616996 | 0.168079799 | 0.585 |
| CpG_Content_50kb | 0.010051874 | 0.011336766 | 0.000702217 | 1.12782613 | 0.069859268 | 0.094883701 | 0.45 |
| **MAF_Adj_ASMC** | -2.35E-14 | -0.617157242 | 0.216968595 | 2.62785E+13 | -9.2385E+12 | 0.000361772 | 0.0228 |
| GTEx_eQTL_MaxCPP | 0.010340409 | 0.040957835 | 0.042048286 | 3.960949016 | 4.066404339 | 0.460675228 | 0.645 |
| **BLUEPRINT_H3K27acQTL_MaxCPP** | 0.016545549 | 0.154033535 | 0.048957806 | 9.309665961 | 2.9589714 | 0.003043716 | 0.0639 |
| BLUEPRINT_H3K4me1QTL_MaxCPP | 0.013372536 | 0.055804441 | 0.040620627 | 4.173063598 | 3.037615968 | 0.301975498 | 0.595 |
| BLUEPRINT_DNA_methylation_MaxCPP | 0.031730971 | 0.071731944 | 0.068873862 | 2.260628684 | 2.170556367 | 0.570875285 | 0.705 |
| **synonymous** | 0.003120031 | -0.11554741 | 0.05464406 | -37.03406001 | 17.51394875 | 0.016635522 | 0.175 |
| non_synonymous | 0.002715412 | 0.077428877 | 0.045669313 | 28.51460088 | 16.81856022 | 0.079402728 | 0.45 |
| Conserved_Vertebrate_phastCons46way | 0.029436054 | 0.132083076 | 0.147149789 | 4.48711892 | 4.998964436 | 0.482751721 | 0.661 |
| Conserved_Mammal_phastCons46way | 0.021439455 | 0.181951912 | 0.144002269 | 8.486778799 | 6.716694462 | 0.256513233 | 0.585 |
| **Conserved_Primate_phastCons46way** | 0.019269072 | 0.412298385 | 0.146052153 | 21.39689926 | 7.579615448 | 0.002659595 | 0.0639 |
| BivFlnk | 0.013549379 | -0.090584176 | 0.071380044 | -6.685485673 | 5.268141534 | 0.129211046 | 0.509 |
| Human_Promoter_Villar | 0.015217007 | 0.021109894 | 0.059607087 | 1.387256638 | 3.917135988 | 0.921387668 | 0.952 |
| Human_Enhancer_Villar | 0.033227431 | -0.031164901 | 0.067740105 | -0.93792688 | 2.038680168 | 0.337092033 | 0.607 |
| Ancient_Sequence_Age_Human_Promoter | 0.004170665 | 0.04201276 | 0.044664683 | 10.07339491 | 10.70924611 | 0.389940307 | 0.635 |
| Ancient_Sequence_Age_Human_Enhancer | 0.005138095 | -0.04291871 | 0.051956773 | -8.353039842 | 10.11206978 | 0.347418827 | 0.608 |
| Human_Enhancer_Villar_Species_Enhancer_Count | 0.066283084 | 0.198473745 | 0.1728375 | 2.994334812 | 2.607565776 | 0.438157956 | 0.635 |
| Human_Promoter_Villar_ExAC | 0.002496662 | 0.007184683 | 0.026444006 | 2.877715547 | 10.591744 | 0.859202907 | 0.902 |

Note: Bold values indicate annotations surviving Benjamini-Hochberg FDR correction at *q*<0.05.

**Table S8. Partitioned SNP heritability in Nonword reading**

| **Category** | **Prop._SNPs** | **Prop._h2** | **Prop._h2_std_error** | **Enrichment** | **Enrichment_std_error** | **Enrichment_p** | **q (FDR-BH)** |
| --- | --- | --- | --- | --- | --- | --- | --- |
| Coding_UCSC | 0.01425914 | 0.112496264 | 0.076096975 | 7.889414404 | 5.336715688 | 0.185427393 | 0.657 |
| Conserved_LindbladToh | 0.024670538 | 0.235441461 | 0.137620173 | 9.543426254 | 5.578320701 | 0.121322968 | 0.643 |
| CTCF_Hoffman | 0.023814664 | 0.098979402 | 0.113087745 | 4.156237543 | 4.748660073 | 0.513687128 | 0.899 |
| DGF_ENCODE | 0.135362603 | 0.253287901 | 0.287174769 | 1.871180777 | 2.121522207 | 0.680196554 | 0.906 |
| DHS_peaks_Trynka | 0.109959825 | 0.210106597 | 0.253693618 | 1.910757834 | 2.307148249 | 0.690010909 | 0.906 |
| DHS_Trynka | 0.165742769 | 0.090012685 | 0.29440968 | 0.543086651 | 1.77630482 | 0.79647442 | 0.981 |
| Enhancer_Andersson | 0.004313758 | 0.007601615 | 0.046903674 | 1.76217899 | 10.87304127 | 0.944263455 | 0.981 |
| Enhancer_Hoffman | 0.041923223 | 0.084861504 | 0.106443055 | 2.024212284 | 2.538999789 | 0.68785386 | 0.906 |
| FetalDHS_Trynka | 0.083682888 | -0.02130211 | 0.218865198 | -0.2545575 | 2.615411626 | 0.627050438 | 0.906 |
| H3K27ac_Hnisz | 0.389043808 | 0.472805711 | 0.098649236 | 1.215301981 | 0.25356845 | 0.403371748 | 0.82 |
| H3K27ac_PGC2 | 0.268521272 | 0.151829998 | 0.171126076 | 0.565430056 | 0.637290575 | 0.488009013 | 0.899 |
| H3K4me1_peaks_Trynka | 0.168870181 | 0.434018892 | 0.237148313 | 2.570133397 | 1.404323197 | 0.2580629 | 0.761 |
| H3K4me1_Trynka | 0.42345507 | 0.61730729 | 0.196651065 | 1.457786986 | 0.46439653 | 0.331713127 | 0.819 |
| H3K4me3_peaks_Trynka | 0.041368465 | 0.211920438 | 0.161019021 | 5.122753195 | 3.892313131 | 0.277408566 | 0.761 |
| H3K4me3_Trynka | 0.132886742 | 0.133100081 | 0.150170077 | 1.001605418 | 1.130060639 | 0.998867491 | 0.999 |
| H3K9ac_peaks_Trynka | 0.038186366 | 0.186373841 | 0.152013558 | 4.880638292 | 3.980833298 | 0.334162731 | 0.819 |
| H3K9ac_Trynka | 0.125302311 | 0.034946371 | 0.168920687 | 0.278896461 | 1.348105118 | 0.581928584 | 0.906 |
| Intron_UCSC | 0.387451333 | 0.373844493 | 0.077023755 | 0.964881163 | 0.198795947 | 0.85877736 | 0.981 |
| PromoterFlanking_Hoffman | 0.008276914 | 0.036460834 | 0.068686378 | 4.405124157 | 8.298549302 | 0.684002872 | 0.906 |
| Promoter_UCSC | 0.046335285 | 0.026604224 | 0.080544006 | 0.574167707 | 1.738286636 | 0.805159548 | 0.981 |
| Repressed_Hoffman | 0.460018094 | 0.414902274 | 0.201160247 | 0.901925989 | 0.437287685 | 0.823070048 | 0.981 |
| SuperEnhancer_Hnisz | 0.167206746 | 0.196364164 | 0.060353974 | 1.174379432 | 0.360954178 | 0.631512964 | 0.906 |
| TFBS_ENCODE | 0.130971511 | -0.2572486 | 0.216219394 | -1.9641569 | 1.650888756 | 0.056151489 | 0.505 |
| Transcr_Hoffman | 0.345192604 | 0.35721795 | 0.192542212 | 1.034836626 | 0.557781974 | 0.950181697 | 0.981 |
| TSS_Hoffman | 0.01780694 | 0.146691162 | 0.082038004 | 8.237864717 | 4.607080392 | 0.115214223 | 0.643 |
| UTR_3_UCSC | 0.01116813 | 0.016584859 | 0.049260074 | 1.485016655 | 4.410771817 | 0.912559607 | 0.981 |
| UTR_5_UCSC | 0.00546018 | 0.010392603 | 0.04278906 | 1.903344551 | 7.836566092 | 0.909048392 | 0.981 |
| WeakEnhancer_Hoffman | 0.020905666 | -0.09802597 | 0.108627208 | -4.68896647 | 5.19606538 | 0.277768499 | 0.761 |
| GERP.NS | 1.745316256 | 2.438610032 | 0.489597649 | 1.397231031 | 0.280520878 | 0.149756156 | 0.643 |
| GERP.RSsup4 | 0.008149925 | -0.04166288 | 0.07699345 | -5.11205677 | 9.447135735 | 0.513947691 | 0.899 |
| **MAFbin1** | 0.102357276 | 0.010270297 | 0.048882548 | 0.10033773 | 0.477567881 | 0.033124985 | 0.417 |
| MAFbin2 | 0.099855414 | 0.024905563 | 0.0564497 | 0.249416251 | 0.565314363 | 0.158207958 | 0.643 |
| MAFbin3 | 0.099606637 | 0.062194103 | 0.044061349 | 0.624397179 | 0.442353549 | 0.390093191 | 0.82 |
| MAFbin4 | 0.100695687 | 0.076125715 | 0.0518549 | 0.755997771 | 0.514966445 | 0.632976514 | 0.906 |
| MAFbin5 | 0.098317123 | 0.095739981 | 0.059445898 | 0.973787455 | 0.604634235 | 0.965287795 | 0.981 |
| MAFbin6 | 0.0996422 | 0.157864127 | 0.053024285 | 1.584309928 | 0.532146874 | 0.257207641 | 0.761 |
| MAFbin7 | 0.099733793 | 0.170509945 | 0.062517934 | 1.709650653 | 0.626848051 | 0.243519284 | 0.761 |
| MAFbin8 | 0.100229335 | 0.083106091 | 0.053081766 | 0.829159363 | 0.529603094 | 0.747010694 | 0.96 |
| MAFbin9 | 0.101099971 | 0.178806761 | 0.059022757 | 1.768613382 | 0.583805876 | 0.163299453 | 0.643 |
| MAFbin10 | 0.098462564 | 0.140477417 | 0.046631837 | 1.426708906 | 0.47359966 | 0.356438898 | 0.82 |
| MAF_Adj_Predicted_Allele_Age | 3.39E-06 | 0.047266821 | 0.220630862 | 13932.99716 | 65036.08909 | 0.827661263 | 0.981 |
| **MAF_Adj_LLD_AFR** | 0.002796474 | -0.3782686 | 0.09628314 | -135.266254 | 34.43019029 | 0.007790361 | 0.282 |
| Recomb_Rate_10kb | 1.552424442 | 2.06611937 | 0.550371046 | 1.330898506 | 0.354523564 | 0.391840922 | 0.82 |
| **Nucleotide_Diversity_10kb** | 4.608695341 | 3.844978407 | 0.302081526 | 0.834287824 | 0.065545996 | 0.008967905 | 0.282 |
| **Backgrd_Selection_Stat** | 0.177719812 | 0.216867407 | 0.019961744 | 1.220277047 | 0.112321435 | 0.013585155 | 0.285 |
| CpG_Content_50kb | 0.010051874 | 0.010782902 | 0.000514085 | 1.072725526 | 0.05114323 | 0.187619411 | 0.657 |
| **MAF_Adj_ASMC** | -2.35E-14 | -0.29680169 | 0.150636005 | 1.26378E+13 | -6.4141E+12 | 0.040823693 | 0.429 |
| GTEx_eQTL_MaxCPP | 0.010340409 | 0.029841653 | 0.039194987 | 2.885925666 | 3.790467595 | 0.613473181 | 0.906 |
| **BLUEPRINT_H3K27acQTL_MaxCPP** | 0.016545549 | 0.120707678 | 0.046051451 | 7.295477333 | 2.783313551 | 0.023403765 | 0.369 |
| BLUEPRINT_H3K4me1QTL_MaxCPP | 0.013372536 | 0.036187465 | 0.041741334 | 2.706103488 | 3.121422547 | 0.582119718 | 0.906 |
| BLUEPRINT_DNA_methylation_MaxCPP | 0.031730971 | 0.058621466 | 0.065229367 | 1.847452621 | 2.055700281 | 0.683608889 | 0.906 |
| synonymous | 0.003120031 | 0.009569146 | 0.051895809 | 3.06700357 | 16.63310763 | 0.900104506 | 0.981 |
| non_synonymous | 0.002715412 | 0.040186914 | 0.038523025 | 14.7995667 | 14.18680911 | 0.338106196 | 0.819 |
| Conserved_Vertebrate_phastCons46way | 0.029436054 | 0.112018171 | 0.13150569 | 3.805475072 | 4.467503988 | 0.530570517 | 0.903 |
| Conserved_Mammal_phastCons46way | 0.021439455 | 0.129089048 | 0.12453334 | 6.021097451 | 5.808605694 | 0.388394476 | 0.82 |
| Conserved_Primate_phastCons46way | 0.019269072 | 0.177860151 | 0.116894481 | 9.230343532 | 6.066430347 | 0.163041495 | 0.643 |
| BivFlnk | 0.013549379 | 0.146828148 | 0.085326083 | 10.83652264 | 6.297416679 | 0.115363113 | 0.643 |
| Human_Promoter_Villar | 0.015217007 | 0.116496823 | 0.060031833 | 7.655698724 | 3.945048561 | 0.097114375 | 0.643 |
| Human_Enhancer_Villar | 0.033227431 | 0.036523063 | 0.065972113 | 1.099184058 | 1.985471361 | 0.960277942 | 0.981 |
| Ancient_Sequence_Age_Human_Promoter | 0.004170665 | 0.038781068 | 0.047176431 | 9.298532295 | 11.31148759 | 0.465560141 | 0.889 |
| Ancient_Sequence_Age_Human_Enhancer | 0.005138095 | 0.001205272 | 0.047331179 | 0.234575589 | 9.211814988 | 0.933790101 | 0.981 |
| Human_Enhancer_Villar_Species_Enhancer_Count | 0.066283084 | 0.198338326 | 0.16882141 | 2.992291772 | 2.546975799 | 0.433992857 | 0.854 |
| Human_Promoter_Villar_ExAC | 0.002496662 | 0.043964295 | 0.026874223 | 17.60922865 | 10.76406092 | 0.124783777 | 0.643 |

Note: Bold values indicate annotations surviving Benjamini-Hochberg FDR correction at *q*<0.05.

**Table S9. Partitioned SNP heritability in phoneme awareness**

| **Category** | **Prop._SNPs** | **Prop._h2** | **Prop._h2_std_error** | **Enrichment** | **Enrichment_std_error** | **Enrichment_p** | q (FDR-BH) |
| --- | --- | --- | --- | --- | --- | --- | --- |
| Coding_UCSC | 0.01425914 | 0.11304896 | 0.12990743 | 7.928175512 | 9.110467126 | 0.42529128 | 0.895 |
| Conserved_LindbladToh | 0.02467054 | 0.19312076 | 0.19809818 | 7.827991449 | 8.029746896 | 0.39426446 | 0.887 |
| CTCF_Hoffman | 0.02381466 | -0.0486073 | 0.17448322 | -2.041064025 | 7.326713594 | 0.67676459 | 0.983 |
| DGF_ENCODE | 0.1353626 | 0.186835 | 0.44737001 | 1.380255678 | 3.304974916 | 0.90836814 | 0.983 |
| DHS_peaks_Trynka | 0.10995982 | 0.0421163 | 0.36901375 | 0.383015367 | 3.355896141 | 0.85371537 | 0.983 |
| DHS_Trynka | 0.16574277 | 0.06289935 | 0.47464895 | 0.379499812 | 2.86376866 | 0.82808444 | 0.983 |
| Enhancer_Andersson | 0.00431376 | -0.0379564 | 0.08244602 | -8.798920512 | 19.11234071 | 0.59998415 | 0.983 |
| Enhancer_Hoffman | 0.04192322 | 0.2184134 | 0.15901357 | 5.209842654 | 3.792970971 | 0.24876725 | 0.681 |
| FetalDHS_Trynka | 0.08368289 | -0.0465519 | 0.33440689 | -0.556289733 | 3.99612033 | 0.69574855 | 0.983 |
| H3K27ac_Hnisz | 0.38904381 | 0.40108803 | 0.15002288 | 1.030958523 | 0.385619502 | 0.93599027 | 0.983 |
| H3K27ac_PGC2 | 0.26852127 | 0.25834358 | 0.28453158 | 0.962097252 | 1.059623966 | 0.97143709 | 0.987 |
| H3K4me1_peaks_Trynka | 0.16887018 | 0.71099979 | 0.43231213 | 4.210333578 | 2.560026477 | 0.18778009 | 0.681 |
| H3K4me1_Trynka | 0.42345507 | 0.6703486 | 0.31343986 | 1.583045397 | 0.740196266 | 0.43501629 | 0.895 |
| H3K4me3_peaks_Trynka | 0.04136847 | 0.34076197 | 0.25417941 | 8.237239834 | 6.144279422 | 0.22027372 | 0.681 |
| H3K4me3_Trynka | 0.13288674 | 0.42451314 | 0.24053535 | 3.194548442 | 1.810077829 | 0.21757772 | 0.681 |
| H3K9ac_peaks_Trynka | 0.03818637 | 0.46541135 | 0.26527212 | 12.18789328 | 6.946775731 | 0.07695066 | 0.636 |
| H3K9ac_Trynka | 0.12530231 | 0.40863634 | 0.23651892 | 3.261203532 | 1.887586288 | 0.20425605 | 0.681 |
| Intron_UCSC | 0.38745133 | 0.44447184 | 0.12017663 | 1.147168167 | 0.310172188 | 0.6326543 | 0.983 |
| PromoterFlanking_Hoffman | 0.00827691 | -0.0422173 | 0.11348819 | -5.100609922 | 13.71141331 | 0.65346557 | 0.983 |
| Promoter_UCSC | 0.04633528 | -0.180541 | 0.15965674 | -3.896403727 | 3.44568371 | 0.11864954 | 0.681 |
| Repressed_Hoffman | 0.46001809 | 0.50082756 | 0.34407881 | 1.088712732 | 0.747967995 | 0.90457705 | 0.983 |
| SuperEnhancer_Hnisz | 0.16720675 | 0.14621418 | 0.09572592 | 0.874451443 | 0.572500336 | 0.82526718 | 0.983 |
| TFBS_ENCODE | 0.13097151 | 0.55375433 | 0.38553702 | 4.228051806 | 2.943670855 | 0.24176745 | 0.681 |
| Transcr_Hoffman | 0.3451926 | 0.51966321 | 0.28173324 | 1.505429725 | 0.816162446 | 0.52808169 | 0.979 |
| TSS_Hoffman | 0.01780694 | -0.0522807 | 0.11876962 | -2.935973953 | 6.669850191 | 0.54852465 | 0.983 |
| UTR_3_UCSC | 0.01116813 | 0.03813103 | 0.08619126 | 3.414271306 | 7.717609033 | 0.75265313 | 0.983 |
| UTR_5_UCSC | 0.00546018 | -0.0008471 | 0.06688589 | -0.155135553 | 12.2497592 | 0.9247691 | 0.983 |
| WeakEnhancer_Hoffman | 0.02090567 | 0.28489334 | 0.16666775 | 13.62756573 | 7.972372347 | 0.09157277 | 0.641 |
| GERP.NS | 1.74531626 | 1.6367143 | 0.80277735 | 0.937775201 | 0.459960967 | 0.89202048 | 0.983 |
| GERP.RSsup4 | 0.00814993 | 0.13966455 | 0.12009544 | 17.13691232 | 14.73577178 | 0.2653299 | 0.696 |
| MAFbin1 | 0.10235728 | 0.10989081 | 0.07110816 | 1.073600398 | 0.694705509 | 0.91568352 | 0.983 |
| MAFbin2 | 0.09985541 | 0.03809969 | 0.08319971 | 0.381548523 | 0.833201795 | 0.44035165 | 0.895 |
| MAFbin3 | 0.09960664 | 0.07767746 | 0.07814191 | 0.779842249 | 0.784505035 | 0.77512744 | 0.983 |
| MAFbin4 | 0.10069569 | 0.08234446 | 0.07236607 | 0.817755536 | 0.718661092 | 0.79916696 | 0.983 |
| MAFbin5 | 0.09831712 | -0.0286353 | 0.07905646 | -0.291254012 | 0.804096577 | 0.07382321 | 0.636 |
| MAFbin6 | 0.0996422 | 0.11092234 | 0.07899874 | 1.113206493 | 0.792824134 | 0.88599428 | 0.983 |
| MAFbin7 | 0.09973379 | 0.09249762 | 0.08648529 | 0.927445131 | 0.867161371 | 0.93318554 | 0.983 |
| MAFbin8 | 0.10022933 | 0.09983915 | 0.09206842 | 0.996107054 | 0.918577614 | 0.99659325 | 0.997 |
| MAFbin9 | 0.10109997 | 0.20867236 | 0.10098759 | 2.06402002 | 0.998888426 | 0.24104843 | 0.681 |
| MAFbin10 | 0.09846256 | 0.20869136 | 0.08617969 | 2.119499571 | 0.875253311 | 0.15508443 | 0.681 |
| **MAF_Adj_Predicted_Allele_Age** | 3.39E-06 | -0.6948935 | 0.31545106 | -204836.0596 | 92986.55335 | 0.03527284 | 0.444 |
| **MAF_Adj_LLD_AFR** | 0.00279647 | -0.5717276 | 0.15335029 | -204.4458761 | 54.83701096 | 0.01111834 | 0.233 |
| Recomb_Rate_10kb | 1.55242444 | 2.90760448 | 0.83026852 | 1.872944281 | 0.534820567 | 0.18190064 | 0.681 |
| **Nucleotide_Diversity_10kb** | 4.60869534 | 3.15414149 | 0.55828986 | 0.684389237 | 0.121138374 | 0.00045737 | 0.0288 |
| Backgrd_Selection_Stat | 0.17771981 | 0.21650373 | 0.03435271 | 1.218230671 | 0.193297035 | 0.1604401 | 0.681 |
| CpG_Content_50kb | 0.01005187 | 0.0108023 | 0.00082047 | 1.07465579 | 0.081623886 | 0.39419421 | 0.887 |
| **MAF_Adj_ASMC** | -2.35E-14 | -0.7467151 | 0.30216832 | 3.1795E+13 | -1.28663E+13 | 0.00121146 | 0.0382 |
| GTEx_eQTL_MaxCPP | 0.01034041 | 0.02804518 | 0.05941445 | 2.712192818 | 5.745851237 | 0.76091988 | 0.983 |
| BLUEPRINT_H3K27acQTL_MaxCPP | 0.01654555 | -0.0388097 | 0.08171419 | -2.345630332 | 4.938741752 | 0.48425621 | 0.94 |
| BLUEPRINT_H3K4me1QTL_MaxCPP | 0.01337254 | -0.0612815 | 0.06102134 | -4.582640759 | 4.563184077 | 0.19852082 | 0.681 |
| BLUEPRINT_DNA_methylation_MaxCPP | 0.03173097 | 0.18398372 | 0.09194113 | 5.798237878 | 2.897520151 | 0.0808235 | 0.636 |
| synonymous | 0.00312003 | 0.11212076 | 0.07981396 | 35.93578513 | 25.5811456 | 0.1348255 | 0.681 |
| non_synonymous | 0.00271541 | 0.00642538 | 0.06513778 | 2.366263206 | 23.98817818 | 0.95431623 | 0.986 |
| Conserved_Vertebrate_phastCons46way | 0.02943605 | 0.00459548 | 0.20343003 | 0.156117396 | 6.910913607 | 0.90236219 | 0.983 |
| Conserved_Mammal_phastCons46way | 0.02143945 | 0.11076147 | 0.20181096 | 5.166244695 | 9.413063998 | 0.65684228 | 0.983 |
| Conserved_Primate_phastCons46way | 0.01926907 | 0.25948967 | 0.18221348 | 13.46664113 | 9.456266553 | 0.15401012 | 0.681 |
| BivFlnk | 0.01354938 | 0.09958575 | 0.12690499 | 7.349838829 | 9.366111673 | 0.49217174 | 0.94 |
| Human_Promoter_Villar | 0.01521701 | 0.02857878 | 0.09208584 | 1.878081432 | 6.051508152 | 0.88441227 | 0.983 |
| **Human_Enhancer_Villar** | 0.03322743 | -0.1713152 | 0.10981438 | -5.155836236 | 3.304931517 | 0.03420978 | 0.444 |
| Ancient_Sequence_Age_Human_Promoter | 0.00417067 | 0.01302524 | 0.06840564 | 3.123059468 | 16.40161393 | 0.89646705 | 0.983 |
| Ancient_Sequence_Age_Human_Enhancer | 0.00513809 | -0.055815 | 0.07086124 | -10.86297391 | 13.79134601 | 0.36919508 | 0.887 |
| Human_Enhancer_Villar_Species_Enhancer_Count | 0.06628308 | -0.1162177 | 0.2083559 | -1.753353394 | 3.143424973 | 0.36889256 | 0.887 |
| Human_Promoter_Villar_ExAC | 0.00249666 | 0.01984787 | 0.04053767 | 7.949761815 | 16.23674689 | 0.6679174 | 0.983 |

Note: Bold values indicate annotations surviving Benjamini-Hochberg FDR correction at *q*<0.05.

Table S10. Pleiotropic loci between ADHD and Word reading

| **SNP** | **CHR** | **BP** | **GENE** | **A1** | **A2** | **mtag_se1** | **mtag_pval1** | **mtag_beta2** | **mtag_pval2** | **p_Shet** | **p_Shom** |
| --- | --- | --- | --- | --- | --- | --- | --- | --- | --- | --- | --- |
| rs1343667 | 6 | 98293830 | RP11-436D23.1 | C | T | 0.002968926 | 8.70E-09 | -0.03720494 | 9.41E-09 | 1.66E-08 | 1.47E-05 |
| rs2107099 | 16 | 17994678 | - | G | A | 0.002985882 | 4.07E-08 | 0.030310419 | 3.44E-06 | 4.42E-08 | 5.20E-06 |
| rs2819340 | 1 | 44039710 | PTPRF | T | C | 0.003515743 | 3.62E-14 | -0.0346223 | 2.65E-06 | 1.46E-15 | 8.12E-13 |
| rs610634 | 18 | 58156977 | - | T | C | 0.003926512 | 3.18E-08 | -0.03680988 | 4.67E-06 | 3.12E-08 | 3.42E-06 |
| rs7506904 | 18 | 50625779 | DCC | G | A | 0.003121451 | 2.26E-09 | 0.031530049 | 2.54E-06 | 1.38E-09 | 2.70E-07 |
| rs78154752 | 14 | 98596553 | RP11-61O1.1 | T | G | 0.003619171 | 2.34E-08 | 0.039850399 | 1.17E-06 | 2.69E-08 | 4.93E-06 |

Table S11. Pleiotropic loci between ADHD and Nonword reading

| **SNP** | **CHR** | **BP** | **GENE** | **A1** | **A2** | **mtag_beta1** | **mtag_se1** | **mtag_pval1** | **mtag_beta2** | **mtag_se2** | **mtag_pval2** | **p_Shet** | **p_Shom** |
| --- | --- | --- | --- | --- | --- | --- | --- | --- | --- | --- | --- | --- | --- |
| rs1343667 | 6 | 98293830 | RP11-436D23.1 | C | T | 0.01649401 | 0.00299092 | 3.49E-08 | -0.0447449 | 0.00903057 | 7.24E-07 | 2.12E-07 | 1.61E-06 |
| rs73163723 | 3 | 1.18E+08 | RP11-384F7.2 | A | G | 0.01904167 | 0.00348537 | 4.67E-08 | -0.051618 | 0.01075256 | 1.58E-06 | 2.58E-07 | 1.68E-06 |
| rs76659130 | 14 | 98596555 | RP11-61O1.1 | C | A | -0.0200745 | 0.00364598 | 3.67E-08 | 0.05327707 | 0.01112091 | 1.66E-06 | 1.97E-07 | 1.29E-06 |
| rs7803385 | 7 | 11501136 | THSD7A | C | T | -0.0289145 | 0.0058506 | 7.73E-07 | 0.08182945 | 0.0176573 | 3.58E-06 | 4.81E-06 | 2.30E-05 |

Table S12. Pleiotropic loci between ADHD and Spelling

| **SNP** | **CHR** | **BP** | **GENE** | **A1** | **A2** | **mtag_beta1** | **mtag_se1** | **mtag_pval1** | **mtag_beta2** | **mtag_se2** | **mtag_pval2** | **p_Shet** | **p_Shom** |
| --- | --- | --- | --- | --- | --- | --- | --- | --- | --- | --- | --- | --- | --- |
| rs10835362 | 11 | 28626786 | - | G | A | 0.02286773 | 0.00305854 | 7.62E-14 | -0.0395588 | 0.00858514 | 4.07E-06 | 1.33E-14 | 1.81E-13 |
| rs11596214 | 10 | 1.06E+08 | SORCS3 | G | A | 0.01755401 | 0.0030297 | 6.87E-09 | -0.0398967 | 0.00851388 | 2.78E-06 | 1.89E-08 | 1.66E-07 |
| rs3850651 | 5 | 88181109 | MEF2C | T | G | 0.01935817 | 0.00303093 | 1.69E-10 | -0.0391949 | 0.00846849 | 3.69E-06 | 1.97E-10 | 2.02E-09 |
| rs6887279 | 5 | 1.04E+08 | RP11-6N13.1 | G | A | 0.02006259 | 0.0035259 | 1.27E-08 | -0.0451411 | 0.00973137 | 3.51E-06 | 3.71E-08 | 2.99E-07 |
| rs7506904 | 18 | 50625779 | DCC | G | A | -0.0185818 | 0.00313047 | 2.92E-09 | 0.04103233 | 0.00862558 | 1.96E-06 | 7.43E-09 | 7.45E-08 |
| rs7613360 | 3 | 49916710 | RP11-6N13.1 | C | T | -0.0173716 | 0.00303093 | 9.96E-09 | 0.03942672 | 0.00856111 | 4.12E-06 | 2.66E-08 | 2.11E-07 |
| rs77960 | 5 | 1.04E+08 | ACTBP13 | G | A | -0.0232704 | 0.00314852 | 1.46E-13 | 0.04442318 | 0.00886433 | 5.40E-07 | 5.91E-14 | 1.39E-12 |

Table S13. Pleiotropic loci between ADHD and Phoneme awareness

| SNP | CHR | BP | GENE | A1 | A2 | mtag_beta1 | mtag_se1 | mtag_pval1 | mtag_beta2 | mtag_se2 | mtag_pval2 | p_Shet | p_Shom |
| --- | --- | --- | --- | --- | --- | --- | --- | --- | --- | --- | --- | --- | --- |
| rs112361411 | 1 | 44183923 | ST3GAL3 | C | T | 0.0204242 | 0.00329211 | 5.51E-10 | -0.045304 | 0.00990337 | 4.77E-06 | 3.97E-10 | 1.82E-09 |
| rs16956411 | 18 | 50775428 | DCC | A | G | 0.01739839 | 0.00309501 | 1.89E-08 | -0.0422619 | 0.00922175 | 4.59E-06 | 2.68E-08 | 1.10E-07 |
| rs216057 | 5 | 88170331 | MEF2C | T | C | 0.01928616 | 0.0030041 | 1.36E-10 | -0.0415882 | 0.0090415 | 4.23E-06 | 7.62E-11 | 3.73E-10 |
| rs2582895 | 11 | 28602173 | - | C | A | 0.02328047 | 0.00311052 | 7.19E-14 | -0.0451186 | 0.00933248 | 1.33E-06 | 1.05E-14 | 7.97E-14 |
| rs4131786 | 16 | 61780558 | CDH8 | A | G | -0.0181131 | 0.00301436 | 1.87E-09 | 0.0436275 | 0.00911808 | 1.71E-06 | 2.08E-09 | 1.12E-08 |
| rs4916723 | 5 | 87854395 | LINC00461 | A | C | -0.0230044 | 0.00301371 | 2.29E-14 | 0.04243569 | 0.00915965 | 3.61E-06 | 2.01E-15 | 1.22E-14 |
| rs7613360 | 3 | 49916710 | ACTBP13 | C | T | -0.0172821 | 0.00305601 | 1.56E-08 | 0.04444523 | 0.00926505 | 1.61E-06 | 2.57E-08 | 1.28E-07 |
